# In vitro and in vivo effects of 2,4 diaminoquinazoline inhibitors of the decapping scavenger enzyme DcpS: Context-specific modulation of SMN transcript levels

**DOI:** 10.1371/journal.pone.0185079

**Published:** 2017-09-25

**Authors:** Jonathan J. Cherry, Christine J. DiDonato, Elliot J. Androphy, Alessandro Calo, Kyle Potter, Sara K. Custer, Sarah Du, Timothy L. Foley, Ariamala Gopalsamy, Emily J. Reedich, Susana M. Gordo, William Gordon, Natalie Hosea, Lyn H. Jones, Daniel K. Krizay, Gregory LaRosa, Hongxia Li, Sachin Mathur, Carol A. Menard, Paraj Patel, Rebeca Ramos-Zayas, Anne Rietz, Haojing Rong, Baohong Zhang, Michael A. Tones

**Affiliations:** 1 Rare Disease Research Unit, Pfizer Worldwide Research and Development, Cambridge, Massachusetts, United States of America; 2 Department of Pediatrics, Feinberg School of Medicine, Northwestern University, Chicago, Illinois, United States of America; 3 Human Molecular Genetics Program, Ann & Robert Lurie Children’s Hospital, Stanley Manne Research Institute, Chicago, Illinois, United States of America; 4 Department of Dermatology, Indiana University School of Medicine, Indianapolis, Indiana, United States of America; 5 Precision Medicine, Pfizer Worldwide Research and Development, Cambridge, Massachusetts, United States of America; 6 Pharmaceutical Sciences, Pfizer Worldwide Research and Development, Groton, Connecticut, United States of America; 7 Primary Pharmacology Group, Pfizer Worldwide Research and Development, Groton, Connecticut, United States of America; 8 Medicine Design, Pfizer Worldwide Research and Development, Cambridge, Massachusetts, United States of America; 9 Pharmacokinetics and Drug Metabolism, Pfizer Worldwide Research and Development, Cambridge, Massachusetts, United States of America; 10 Business Technology, Pfizer Worldwide Research and Development, Cambridge, Massachusetts, United States of America; 11 Clinical Genetics, Pfizer Worldwide Research and Development, Cambridge, Massachusetts, United States of America; Universitatsklinikum Wurzburg, GERMANY

## Abstract

C5-substituted 2,4-diaminoquinazoline inhibitors of the decapping scavenger enzyme DcpS (DAQ-DcpSi) have been developed for the treatment of spinal muscular atrophy (SMA), which is caused by genetic deficiency in the Survival Motor Neuron (SMN) protein. These compounds are claimed to act as *SMN2* transcriptional activators but data underlying that claim are equivocal. In addition it is unclear whether the claimed effects on *SMN2* are a direct consequence of DcpS inhibitor or might be a consequence of lysosomotropism, which is known to be neuroprotective. DAQ-DcpSi effects were characterized in cells *in vitro* utilizing DcpS knockdown and 7-methyl analogues as probes for DcpS vs non-DcpS-mediated effects. We also performed analysis of *Smn* transcript levels, RNA-Seq analysis of the transcriptome and SMN protein in order to identify affected pathways underlying the therapeutic effect, and studied lysosomotropic and non-lysosomotropic DAQ-DCpSi effects in 2B/- SMA mice. Treatment of cells caused modest and transient *SMN2* mRNA increases with either no change or a decrease in *SMNΔ7* and no change in *SMN1* transcripts or SMN protein. RNA-Seq analysis of DAQ-DcpSi-treated N2a cells revealed significant changes in expression (both up and down) of approximately 2,000 genes across a broad range of pathways. Treatment of 2B/- SMA mice with both lysomotropic and non-lysosomotropic DAQ-DcpSi compounds had similar effects on disease phenotype indicating that the therapeutic mechanism of action is not a consequence of lysosomotropism. In striking contrast to the findings *in vitro*, *Smn* transcripts were robustly changed in tissues but there was no increase in SMN protein levels in spinal cord. We conclude that DAQ-DcpSi have reproducible benefit in SMA mice and a broad spectrum of biological effects *in vitro* and *in vivo*, but these are complex, context specific, and not the result of simple *SMN2* transcriptional activation.

## Introduction

Spinal Muscular Atrophy (SMA) is an inherited, autosomal recessive neuromuscular condition and a common genetic cause of mortality in infants and children. The disease is characterized by loss of function and ultimately degeneration of lower motor neurons whose cell bodies are located in the ventral horn of the spinal cord. At a genetic level SMA is caused by deletion or (less commonly) other loss-of-function mutations in the survival of motor neuron 1 (*SMN1*) gene [1, 2]. Duplication and inversion of a region of chromosome 5q13 encompassing this and several other genes occurred in *H*.*sapiens’* distant evolutionary past but the acquisition of the characteristic nucleotide differences between *SMN1* and *SMN2* occurred only since the divergence of chimpanzees and man from their common ancestor [3]. A C/T substitution in exon 7 of *SMN2* disrupts an exon splicing enhancer sequence with the result that the majority of transcripts produced from this gene are alternatively spliced, missing exon 7, and produce a truncated, unstable SMN protein. Nevertheless, a significant fraction of *SMN2* transcripts are full length and encode a fully functional 294 amino acid SMN protein identical to that produced from the *SMN1* gene. For this reason, and because it exists in the population in variable copy number, *SMN2* functions as a disease modifier gene such that higher copy number tends to be associated with milder disease [4]. All SMA patients have at least one copy of *SMN2*, presumably because complete absence of SMN protein is embryonic lethal in humans, as is the case in mice [5].

The presence of the *SMN2* gene in all SMA patients provides an attractive opportunity for therapeutic approaches aimed at increasing the amount of full length SMN protein produced from this gene. This philosophy underpinned the use of a *SMN2* promoter β-lactamase (βLAC) reporter gene assay to screen a library of over half a million compounds for transcriptional activators [6]. After hit confirmation, removal of false positives due to fluorescence, and dose-response determination, this effort resulted in 17 unique compounds belonging to 9 different structural scaffolds, one of which (C5-substituted 2,4 diaminoquinazolines, DAQ) formed the basis for medicinal chemistry optimization using the βLAC assay. This effort resulted in two compounds that have demonstrated efficacy in 3 different mouse models of SMA [7–9]. In parallel, a protein microarray experiment identified the decapping scavenger enzyme DcpS as a binding partner of these compounds, which bind to the DcpS active site and inhibit catalytic activity with a potency correlated with their activity in the βLAC assay [10]. These findings led to the proposal that DcpS inhibition is responsible for the activity of these compounds in the βLAC assay. The mechanism(s) whereby DcpS inhibition could result in an apparent increase in *SMN2* promoter activity have been speculated as being secondary to an accumulation of methylated nucleotide levels causing sequestration of cap binding proteins and thereby inhibition of one or more of the cap-dependent processes such as pre-mRNA splicing or 5’-3’ exonucleolytic decay. However, direct evidence to support such a mechanistic link between DcpS and *SMN2* promoter activity is lacking. Furthermore, the evidence that the therapeutic effect of such diaminoquinazoline DcpS inhibitor (DAQ-DcpSi) compounds is the result of elevation of SMN is equivocal. It has been reported that compound 11a (subsequently designated as D156844) elevated the endogenous mouse *Smn* mRNA in NSC-34 cells, raised SMN protein levels and dramatically increased the numbers of SMN-positive nuclear foci (gems) in SMA patient fibroblasts [11]. However, when dosed to SMA Δ7 mice, the same compound had no statistically significant effect on either *SMN2* or *SMN*Δ7 transcript levels or SMN protein in CNS tissues, nor did it have any statistically significant effect on snRNP assembly, a function of the Smn complex that is defective in SMA [9]. More recently [8] the compound RG3039 (also known as D157495) was shown to improve survival, motor function, neuromuscular junction architecture and electrophysiology in 2B/- SMA mice, causing profound and prolonged inhibition of tissue DcpS activity. However these beneficial effects also occurred in the absence of any change in SMN protein levels in the CNS or any of the peripheral tissues studied. In contrast, in the same study the SMN-containing nuclear bodies known as Gems were reported to increase in the nuclear compartment of choline acetyltransferase-positive neurons from the spinal cord. A similar study did report modest but statistically significant elevations of both full length and Δ7 *SMN* transcripts in SMAΔ7 mice treated with RG3039, but no detectable change in SMN protein or snRNP assembly [7]. Therefore the assumption based on their mode of discovery that DAQ-DcpSi exert their therapeutic benefit in SMA mice via positive modulation of SMN is not consistently supported by the totality of the evidence, nor is it clear what effects may be due to DcpS inhibition *per se* versus other properties of the compounds such as lysosomotropism.

Therefore the current studies were undertaken to provide a comprehensive survey of the effects of DAQ-DcpSi in cells, study the role of DcpS inhibition and test whether non-lysosomotropic DAQ-DCpSi have beneficial effects in SMA mice.

## Materials and methods

### Synthesis of DAQ-DcpSi and their 7-methyl derivatives and *in vitro* DcpS inhibition assay

A separate report describes the synthesis of novel diaminoquinazoline compounds [12]. The inhibitory potency of compounds was performed in an assay volume of 20 μl containing 0.05 nM recombinant human DcpS, varying amounts of compound in a buffer of composition 50 mM Tris, 20 mM MgCl_2_, 60 mM (NH_4_)_2_SO_4_, pH 7.9. Incubations were initiated by the addition of 50 nM m7GpppA-biotin substrate and proceeded for 45 min at room temperature before being terminated by the addition of 1 μM D156844 [11]. Production of ADP-biotin was quantitated by transferring 18 μl of the quenched reaction (or biotin-ADP standard) to a streptavidin-coated plate (Pierce), incubating for 2 hours then washing 3 times followed by detection using an anti-ADP mouse antibody (Bellbrook lab) and an HRP-conjugated Goat anti-mouse IgG (Invitrogen).

### Cell culture

SMA patient lymphoblasts (GM23689 or GM23686, Coriell Institute biorepository) were grown in Advanced RPMI supplemented with 2% FBS and penicillin-streptomycin at 37°C in 5% CO_2_. Cells are maintained in suspension at a density of between 200,000–1,000,000 cells per mL. For drug treatment, cells were plated in 24-well dishes at 500,000 cells per well (1 mL at 0.5 million cells per mL). 1μL of each compound or DMSO was added to each well (0.1% DMSO final) and incubated for 24 hours. SMA patient and carrier-derived fibroblasts (GM03813 and GM03814, respectively, Coriell Institute biorepository) were grown in Cascade 106 media supplemented with low serum growth supplement (LSGS) at 37°C in 5% CO_2_. For drug treatment, cells were plated in 6-well dishes at 20,000–40,000 cells per well. Two wells were collected for each data point and pooled to generate a single lysate. Compounds were added as appropriate to reach desired final concentration and incubated for 72 hours or 6–7 days with fresh media and compound added every 24 hours. Neural progenitor cell (NPC) cultures (GM24468, Corriell Institute biorepository) were generated and maintained according to Reinhardt et al [13]. For treatment, NPCs were plated on geltrex coated 6-well dishes at a density of 60,000 cells per cm^2^ then incubated overnight. Compound or DMSO was included as appropriate to reach the desired concentration (final DMSO of 0.2%) with fresh media every 24 hrs. HEK-293T (ATCC- CRL-3216) and Hela-S3 (ATCC CCL-2.2) cells were grown as described for HEK-293T below.

### Knockdown experiments

HEK-293T cells were grown in DMEM high glucose Glutamax (Life Technologies #10569010) supplemented with 10% FBS and penicillin-streptomycin at 37°C in 8% CO_2_. For lentivirus infection by spinoculation, 50,000 cells in growth DMEM were mixed with 1 μL of polybrene at 8 mg/mL and 100 μL of virus at 1 x 10^6^ TDU/mL in a final volume of 1 mL. This gave an MOI of 2. The samples were spun at 800 xg for 30 minutes at RT. Media was removed and cells were replated in 48 well dishes and incubated over the weekend. Puromycin was used for 4 days at 2 mg/mL to select for stable cell lines. Lentiviruses were from Sigma MISSION collection and in the pLKO.1 vector -TRC version 2 -Puro marker (details provided in S1 Table). For compound treatment studies, cells were plated in 6-well dishes at 300,000 cells per well and incubated overnight. The cells were then treated for 24 hours with DMSO or RG3039 at a final concentration of 10 nM and 100 nM.

### Reporter gene assays

The *SMN2* reporter cell line [14, 15] was cultured in high-glucose DMEM supplemented with 10% (v/v) fetal bovine serum (FBS) and 1X Penicillin/Streptomycin. Reporter cells media was supplemented with hygromycin during maintenance. All cells were maintained at 37°C and 5°C CO_2_. After seeding in 96-well white tissue culture plates at 25,000 per well, cells were incubated overnight before being exposed to different doses of compounds or DMSO for 24 hrs. The final DMSO concentration was kept constant at 0.1% (v/v). Firefly and renilla luciferase expression were assayed with DualGlo (Promega E2920) and measured on a PHERAstar FS microplate reader (BMG Labtech). Relative light units are normalized to DMSO control and expressed as a percentage.

### Western blot analysis

Cell pellets were lysed in lysis buffer (50 mM Tris pH.7.5, 150 mM NaCl, 0.5% NP-40, 6M Urea, and protease inhibitor). Lysates were incubated at 95°C for 5 minutes and cleared at 14,000 RPM for 15 minutes. Cleared lysates were quantified by BCA assay. A total of 5 μg of each lysate was mixed with NuPage LDS sample buffer with 100uM DTT and loaded and separated on a NuPage 10% Bis-Tris gel. Proteins were transferred to 0.2 micron nitrocellulose using the BioRad Turbo Transfer system. Protein bands were visualized using HPA039632 mouse anti-DcpS (Sigma Prestige), 2F1 mouse anti-SMN1(Cell Signalling), and DM1a mouse anti-alpha tubulin (Cell Signaling) antibodies and imaged and quantified using the GE Healthcare ImageQuant LAS4000.

### Cell experiments: RNA isolation, quantitation, cDNA synthesis, and analysis by ddPCR

Cell pellets were lysed in RLT and the RNA was isolated using RNeasy Mini Kit from Qiagen with modifications to the protocol. 700μl of RPE buffer was used instead of 500μl and the columns were mixed by tilting to wash the leftover RW1 Buffer from the cap. RNA was quantified using the NanoDrop 8000 UV/Vis spectrophotometer and confirmed using the Readiplate 96 RiboGreen RNA quantification kit. A260/A230 ratios were typically 1.8 or above. 500 ng RNA was used to synthesize cDNA using the iScript cDNA synthesis kit (BioRad). The cDNA was diluted to 5 ng/μL. Taqman assays were set-up using 5 μL of cDNA (25 ng of total RNA) with the 2x droplet PCR Supermix (BioRad). Droplets were generated using the QX200 Droplet Generator (BioRad) and a two-step PCR reaction (95°C for 5 min; 95°C for 30 sec and 59°C for 1 min for 40 cycles; 98°C for 10 min; and 4°C hold). Droplets were read on the QX200 Droplet reader and quantified using the Quantasoft software (BioRad). Details of primer/probe sets are provided in supplementary data (S2 and S3 Tables).

### Preparation of RNA, miRNA, cDNA library preparation and RNA-Seq profiling of N2a cells treated with RG3039 or PF-06738066

N2a cells (ATCC- CCL-131) were cultured in DMEM high glucose Glutamax (Life Technologies #10569010) supplemented with 10% FBS and penicillin-streptomycin at 37°C in 5% CO_2_. 3 x 10^6^ cells were plated in 10 cm dishes (4 replicates per condition) and incubated overnight before being treated for 24 hours with 1 μM RG3039, 1 μM PF-06802336, or DMSO vehicle control. Cells were then pelleted and lysed in RLT and the RNA was purified according to the protocol for the Qiagen miRNeasy kit to separate and enrich small RNA and large (>200 nt) fractions separately. Large and small RNA was quantified using the NanoDrop 8000 UV/Vis spectrophotometer and large RNA quality was confirmed using Bioanalyzer (Aglient). RNA quality was also validated using qPCR for DcpS responsive genes. RNA fractions were used for cDNA library preparation which was performed using Truseq mRNA v2 reagents or TailorMix miRNA reagents (RS-122-2101 Illumina, San Diego CA, USA and TM302 Seqmatic, Fremont CA, USA). Libraries were run on a Nextseq 500 (Illumina) using a paired-end run (2x75bp) generating ~60 million reads per library for the mRNA-seq and 1x75bp generating ~5 million reads per library for the miRNA-seq. Demultiplexing, alignment, and differential analysis for the mRNA-seq were performed as described below. For the miRNA alignment, briefly the sequences between 16 and 35 nucleotides with average phred score ≥ 30 were retained for analysis. The reads were first mapped to mouse miRNAs using Bowtie [16] without allowing for mismatches. Instead of discarding the remaining reads, we explored the possibilities of RNA editing to include potential isomiRs [17]. These reads were mapped to miRNAs that have at most 1 base mismatch, a substitution at the 5’ or 3’ ends, or have 1 or 2 extra bases at either ends. Although 1 base mismatch would include 1 base substitution/deletion/addition at the ends, we made sure that 1 base mismatch was inside the miRNA and that all categories were mutually exclusive. Reads mapping to protein coding exons and other small RNAs were removed prior to mapping to miRNAs. Reads that uniquely mapped to miRNAs were considered and were profiled as: 1) identical to miRNAs, 2) perfect match to miRNAs but are of shorter length (no more than 5 bases short), 3) mapping to miRNAs with 1 substitution, 1 or 2 extra bases at 3’ and 5’ ends and with those with at most 1 mismatch (occurring inside the sequence and not on ends), 4) mapping to the precursor miRNA. Thus, steps 2–4 constitute “IsomiRs”-alternative forms of the mature miRNAs. We merged the counts of reads identical to a miR and those mapping to isomeric forms as they are found to have identical/very similar functions [18]. The unaligned reads were mapped to the mouse genome and an internally developed database of rRNAs from bacteria, fungi, plant and eukaryotes to detect presence of contaminants. For differential analysis of miRNAs, only those with counts of ≥5 in at least 1/3^rd^ samples (treatment vs control) were considered for analysis. The counts were normalized and analyzed using EdgeR [19] packages in R-Bioconductor. The *P*-values were corrected by FDR for multiple testing and miRNAs with FDR ≤15% and |FC|>1.5 were considered to be differentially expressed.

### SMA mouse studies

This study was carried out in strict accordance with the recommendations in the Guide for the Care and Use of Laboratory Animals of the National Institutes of Health. The protocol was approved by the Committee on the Ethics of Animal Experiments of the Anne and Robert H. Lurie Children’s Hospital (IACUC Number: 16–005). No special training in animal handling or care was required for these studies and all efforts were taken to limit any potential animal distress as described for housing and humane functional endpoints noted below. Personnel conducting studies followed the approved standard operating procedures for daily monitoring, dosing, weaning and euthanasia according to the approved study protocol. All adult mice were monitored at least weekly for health and housed in a pathogen-free controlled animal facility, fed *ad libitum* water and food (chow NIH-31) with a 12 hr light:12 hr dark photoperiod. Smn+/- mice in an FVB/N congenic background were obtained from the Jackson Laboratory, Bar Harbor, Maine (strain # 6214). Line maintenance was maintained by interbreeding of the congenic line and every 4th generation mice were outbred to wild type FBV/N mice (jax # 001800) to reduce the possibility of new mutations within the line. Smn 2B mice were internally developed and maintained in the DiDonato laboratory. To generate the SMA mice used in this study an intercross between Smn 2B/2B and Smn +/- mice was performed. This generated litters in which 50% of the offspring were control (2B/+) and 50% SMA (2B/-). The day of birth was considered to be P0. If pups were born after 3pm, the following day was considered P0. At P1, pups were counted. If greater than 10 pups to a cage, pups were randomly chosen and moved to another cage to increase litter size, culled to reduce litter size or genotyped at P1 and culled at P2 to reduce litter size to 10 or fewer pups. Thereafter, no culling of pups was performed for drug studies. Individual mice were genotyped from DNA obtained from tail snips by polymerase chain reaction (PCR). All mice were weaned at P21 and separated into male and female cages. In total, 412 mice were used (132 mice for single dose PK analysis of adult wild type (Smn+/+) and P13 2B/+ and 2B/- with the results reported in supplemental file 1; 205 mice were generated for the survival study and 115 were generated to be used for PK and tissue harvest from P4-P16 drug dosing). All compounds were provided to the investigator as pre-prepared dosing solutions once/week during study. The investigator was blinded to compound/vehicle identity. The vehicle was 2% Polyvinyl pyrrolidone/1% Poloxamer (Pluronic F108) and the drugs PF-06687859 (RG3039) and PF-06738066 (nanosuspension) were prepared in the same. Dosing solutions were prepared to deliver the indicated dose using a dosing volume of 2.5 μL/g body weight. For the PK single dose studies a single ip injection was used. For survival studies drug level and tissue harvest mice from P4-P16 treatment, mice were treated twice daily (b.i.d.) via intraperitoneal (i.p.) injection starting on P4 and continuing until P20 or the sacrifice of the animals, whichever occurred sooner. The dosing interval was 12 hours ± 90 minutes. Body weights were recorded daily in the AM and dosing volume was adjusted accordingly. The survival and multi-day drug dosing analysis study was conducted in two parts. The first part was a survival study to investigate the effect of investigational drugs on survival and motor function in SMA (2B/-) mice in a FVB/N genetic background. Three investigational drugs (PF-06687859 (RG3039), Vehicle and PF-06738066 were provided in coded vials and administered to SMA and control mice from postnatal day 4–20 (P4-P20). A 4^th^ cohort, control mice (2B/+), received vehicle as a study control. Functional assessment began at P10 and every other day thereafter using a 55° negative geotaxis/climb test. Mice were placed facing the bottom of the incline and their ability to reorient themselves facing up the incline, and their ability to climb to the top of the incline within 1 minute were assessed. Mice were weaned at P21 and the study was terminated at P35 with euthanasia of any surviving animals. Survival was monitored daily from P2-P4 (AM); P4-P20 twice daily at dosing times; P21-P35 at least once per day at time of weight (AM) or twice on those days (AM and PM) in which incline plane testing occurred. At P4, litters were randomized across treatment arms to uniformly control litter size and litter weight as appropriate. Litter size entry was a minimum of 6 and maximum of 10. Litters with average weights at P4 that were greater or less than two stand deviations below the average P4 litter weight of untreated litters were also excluded from study entry. All mice within a litter were dosed with only one test article or received no dose at all. Dosers/phenotypic testers were blinded to the test articles being administered. At P21, all mice were weaned and followed for survival, weight and function until the study endpoint at P35. A small group of untreated SMA mice (siblings to vehicle treated controls) were also followed in this study to ensure the baseline survival of untreated SMA mice did not differ from prior work [8]. All SMA mice weaned at P21 received 1 nesting pad and at least 1 control mouse in their cage. Additionally, each SMA cage was also provided with water soaked food pellets that were ground to a watery paste (porridge) and placed in the cage bottom. Cage bottoms were changed 3X /week (M, W, F). Additional food porridge was provided as needed between cage bottom changes. Mice meeting humane functional endpoints (30 percent loss of weight for two consecutive days and/or an inability to right within 1 minute or obvious state of distress) were immediately euthanized with CO_2_ followed by cervical dislocation as a secondary measure. In total, 29 litters were born that corresponded to 205 mice (PF-06687859 [RG3039] used 5 litters, 20 SMA mice [12 male/8 female]; vehicle used 5 litters, 21 SMA mice [11male/10 female], PF-06738066 used 7 litters, 21 SMA mice [11male/10 female] and vehicle control 2B/+ treatment cohort used 5 litters, 24 2B/+ control mice (12 male/12 female). The other litters did not fulfill study entry criteria and were euthanized. There were 29 SMA mice across these cohorts that did not reach the P35 study endpoint, presumably dying from disease manifestation, and of these, only 1 SMA mouse at time of cage observation met the humane functional endpoint as described above. For the second part of the study, animals were dosed from P4-P16 using the same dosing regimen as described above, but 12 hours following the last dose were euthanized and tissues harvested for analysis of drug levels, DcpS activity, RNA and protein analysis as follows. No mice died prior to P16 harvest and a total of 96 mice were used for this portion of the study from the 115 that were generated from breeding. Those not used for analyses were also euthanized using CO2 inhalation followed by cervical dislocation as a secondary measure.

### RNA preparation from mouse tissues for *Smn* transcript analysis by ddPCR

Skeletal muscle, spinal cord, and liver were harvested from treated or untreated SMN 2B/- or 2B/+ mice. Samples for RNA isolation were frozen in RNALater (Qiagen). Sample for protein analysis were flash frozen in liquid N_2_. Spinal cord and liver RNA was extracted and purified according to the protocol for the RNAeasy (Qiagen). Skeletal muscle RNA was extracted and purified using the Fibrous Tissue RNAeasy mini kit. All RNA was quantified using the NanoDrop 8000 UV/Vis spectrophotometer and large RNA quality was confirmed using Bioanalyzer (Agilent). RNA analysis by ddPCR was performed as described above.

### RNA-Seq analysis on spinal cord tissue from 2B/- and 2B/+ mice treated with RG3039, PF-06738066 or vehicle

Six animals from each condition were selected at random for preparation of RNA from spinal cord tissues. Total RNA was extracted with miRNeasy kits (217004, Qiagen, Valencia, CA) and 1 μg of RNA was used for cDNA library preparation which was performed at Seqmatic (Fremont, CA) using Illumina (San Diego, CA) Truseq mRNA v2 reagents (RS-122-2101, Illumina, San Diego, CA, USA). Libraries were run on a Nextseq 500 (Illumina) using a paired-end run (2x75bp) generating ~60 million reads per library and demultiplexed using the Bcl2fastq algorithm (Illumina, San Diego, CA). RNA-Seq alignment and differential analysis were performed using open source tools implemented as an in-house pipeline as follows: The mouse genome (mm10) and gene annotation were used for mapping and read counting. Reads were mapped using STAR v2.4.0h [20] on the Pfizer high performance computing cluster and read counts were performed utilizing featureCounts [21]. Genes with expression less than 1 CPM were considered low expression and if a gene had no or low expression across all sample it was omitted from further analysis. Differential analysis was performed using the R package edgeR 3.8.5 [19] and all genes with a Benjamini-Hochberg *P*-value less than 0.05 were reported as differentially expressed.

### Ingenuity pathway analysis

The differentially regulated gene lists for both the spinal cord mRNA-seq and the N2a mRNA-seq results with a corrected *P*-value less than 0.05 were loaded into IPA and analyzed against the Ingenuity Knowledgebase, Ingenuity Expert Information, and with experimentally observed confidence.

### Western blot analysis of mouse tissues

All tissues were resuspended in RIPA buffer supplemented with cOmplete EDTA-free protease inhibitor (Roche) and PhosStop phosphatase inhibitor (Roche). Tissues were homogenized using the TissueLyzer (QIAgen) at a frequency of 30 Hz for 30 seconds (liver and spinal cord) and 1 minute (skeletal muscle). Protein gel electrophoresis and Western blot transfer were performed as described above for cells using 10 μg of tissue lysates. Mouse monoclonal 8/SMN (BD, 610647) and rabbit polyclonal anti-alpha tubulin (abcam, ab15246) were used for protein detection and bands were imaged and quantified in a LI-COR Odyssey CLX system.

### Statistical analysis

Except where specified otherwise, statistical analysis was performed using GraphPad Prism software (version 6.0). Kaplan-Meier survival curves were generated from the data and analyzed using Mantel-Cox or Gehan-Breslow-Wilcoxon tests. The motor function task of combined 55° negative geotaxis and climb test were analyzed using a Fisher’s Exact test. Determination of statistical significance for normally distributed datasets was performed using student’s unpaired T-test in GraphPad Prism; for non-normally distributed datasets, the Mann-Whitney test was used when comparing two groups or one-way analysis of variance (ANOVA) with a Bonferroni post hoc comparison if comparing against multiple groups. RNA-seq stastistical analyses are described within that section. For all studies, *P*< 0.05 was considered significant.

## Results

### Characterization of 7-methyl analogues of C5-substituted 2,4 diaminoquinazolines as probes of DcpS inhibition-dependent effects

**Fig 1:**
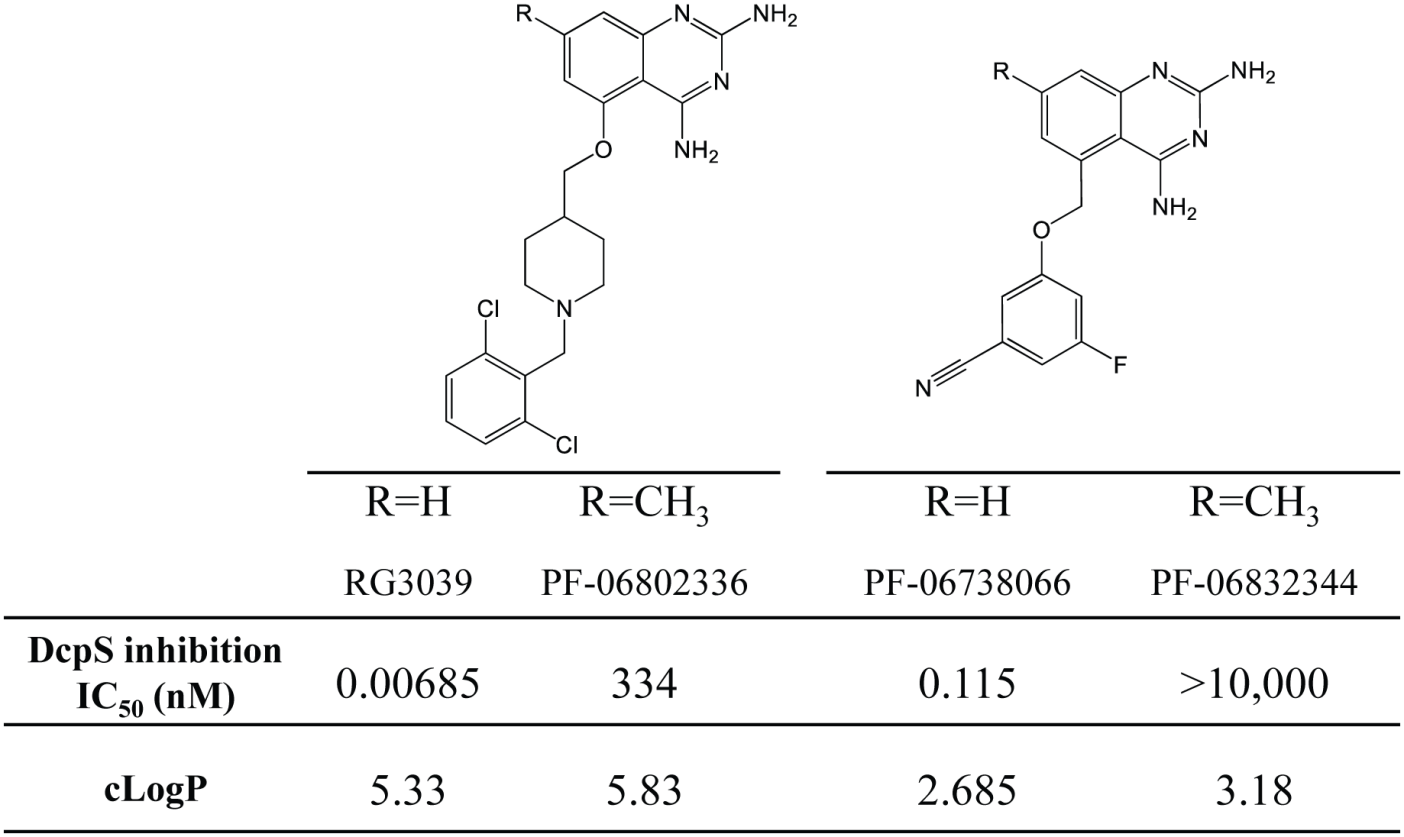
The chemical structures of C-5-substituted 2,4-diaminoquinazoline DcpS inhibitors used in this study. The active DAQ-DcpSi compounds are RG3039 and PF-06738066 and their inactive 7-methyl analogues are PF-06802336 and PF-0683234, respectively. DcpS inhibition activity (IC_50_) and cLogP for each compound are provided. Values represent the mean of two or more independent assays.

To generate experimental tools to probe those effects of DAQ-DcpSi resulting from DcpS inhibition, we designed and synthesized the 7-methyl analogues of several potent DcpS inhibitors based on co-crystal structures of DcpS and DAQ-DcpSi [12]. As predicted, these were found to be more than a thousand-fold less active as inhibitors of purified DcpS than the parent molecules (Fig 1). Therefore we utilized these 7-methyl analogues in further experiments as a means of distinguishing the effects of compounds that were chemotype-driven from those due to intrinsic DcpS inhibition with the objective to identify and validate DcpS-sensitive genes (see below).

### Selection of reference transcript for use in droplet digital PCR

As previously reported effects of DAQ-DcpS on *SMN* transcripts have been modest in magnitude and not necessarily statistically significant, we chose to use droplet digital PCR (ddPCR) as the most appropriate methodology to detect and quantitate any small changes occurring. Initial data obtained from cells treated with DAQ-DcpSi were significantly noisy which we speculated might be improved by employing more stringent RNA quality criteria. As the ddPCR method is exquisitely sensitive, being capable of detecting a change of as little as 20% or less in a given transcript, it is therefore also likely to be highly sensitive to variations in salt content of RNA samples, which is known to inhibit the reverse transcription step in cDNA preparation. We found that by re-purifying RNA, there was a significant improvement in the data quality and consistency. *Proteasome 26S Subunit*, *Non-ATPase 14* (*PSMD14*) was chosen as the reference gene for comparing the expression level of all transcripts reported herein because unlike a number of other commonly used housekeeping genes that were considered, it was clearly unaffected in cells exposed to DAQ-DcpSi treatment in any experiment. With these technical improvements, we were able to obtain extremely consistent data with very low inter-replicate variation.

### Validation of DAQ-DcpSi-sensitive transcripts: DPM3 and PAQR8 are DcpS-sensitive genes

**Fig 2:**
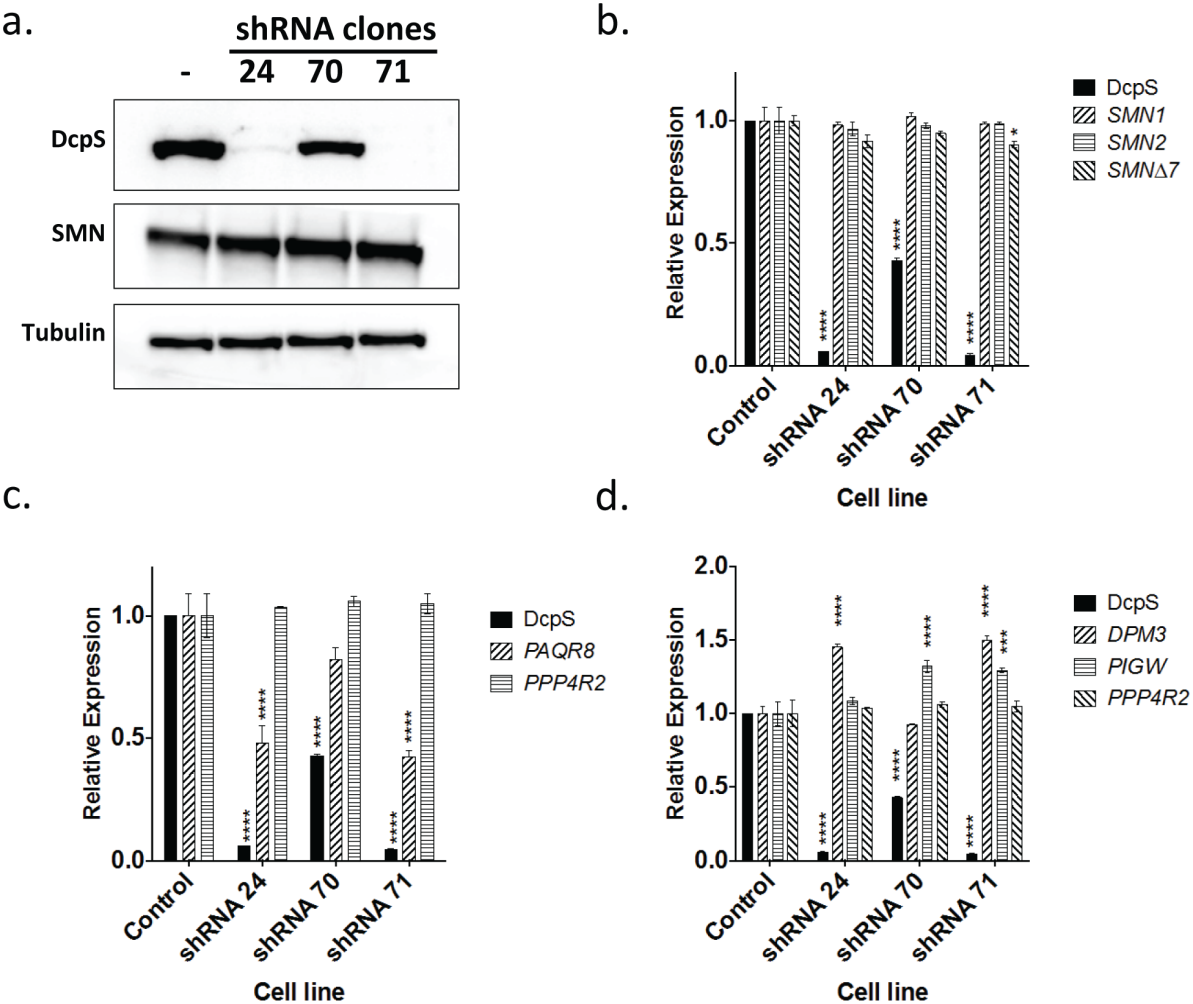
Effect of DcpS knockdown on putative DcpS-sensitive genes in stable clones of lentivirus-infected HEK-293 cells. Western blot (A) and transcript (B) analysis of DcpS lentiviral HEK293T knockdown clones. C. and D., Transcripts of putative DcpS-sensitive genes. All numerical data are shown as mean ± s.e.m. and where not shown error bars are within the size of the symbols. Levels of statistical significance were noted as *P*<0.05 (*), *P*<0.01(**), *P*<0.001(***), *P*<0.0001(****).

**Fig 3:**
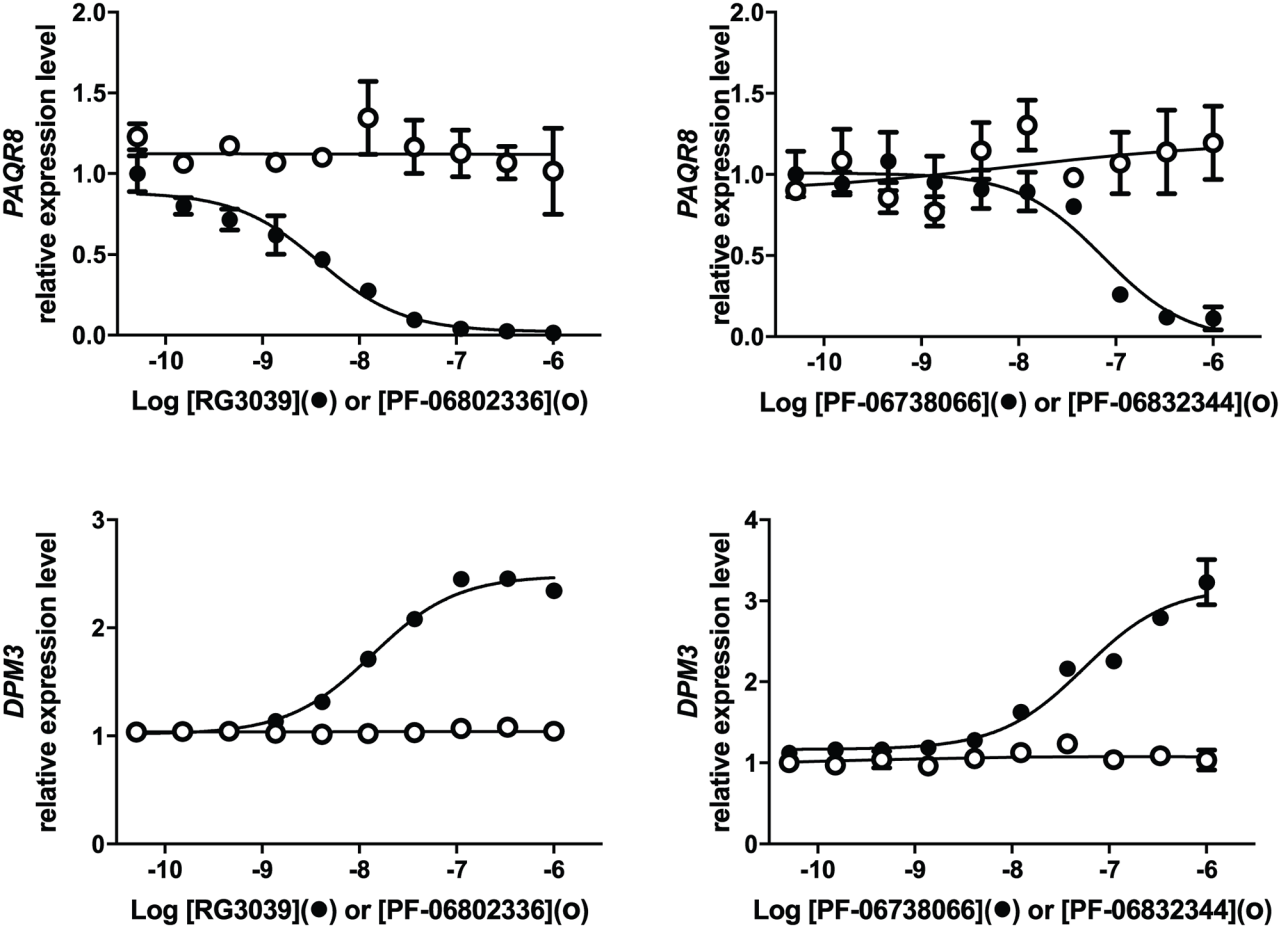
Effect of active DAQ-DcpSi and their 7-methyl analogues on *PAQR8* and *DPM3* transcripts. SMA-derived lymphoblasts were treated with active DAQ-DcpSi (RG3039, PF-06738066) or their 7-methyl analogues (PF-06802336 and PF-06832344 respectively) and the levels of *PAQR8* and *DPM3* transcripts measured using ddPCR. All data shown as mean ± s.e.m. and where not shown error bars are within the size of the symbols. Significant P-values were denoted as *P*<0.05 (*), *P*<0.01(**), *P*<0.001(***), *P*<0.0001(****).

A number of gene transcripts have been observed to change in response to DAQ-DcpSi [22]. Thus we sought to determine whether some of these were responsive to DcpS inhibition *per se* or some other effect(s) of these compounds unrelated to DcpS. Knockdown experiments were performed by infecting HEK-293T cells with DcpS shRNA lentiviral vectors and isolating puromycin-resistant clones for analysis (Fig 2). Successful knockdown of *DcpS* mRNA and protein was confirmed by ddPCR and Western blot and was almost complete in clones 24 and 71 but only partial in clone 70 (Fig 2A). No changes in *SMN* transcripts were observed in any clone (Fig 2B). Testing the DcpS-sensitive transcripts reported by Zhou *et al*. (2015) [22], the pattern of changes in Dolichyl-Phosphate Mannosyltransferase Subunit 3(*DPM3*) and Progestin and AdipoQ Receptor Family Member 8 (*PAQR8*) was consistent with their being DcpS-sensitive genes, both qualitatively (same direction of change as induced by pharmacological DcpS inhibition) and quantitatively (because the degree of modulation was related to the degree of DcpS knockdown). The lack of effect in clone 70 suggests that greater than 50% loss of DcpS activity is required to cause a change in either *DPM3* or *PAQR8*. The data did not validate Phosphatidylinositol Glycan Anchor Biosynthesis Class W (*PIGW*) or Protein Phosphatase 4 Regulatory Subunit 2 (*PPP4R2*) as being DcpS-sensitive genes (Fig 2C and 2D). In addition to these knockdown data, 7-methyl substitution ablated the ability of DAQ compounds to change the expression of *PAQR8* and *DPM3* in SMA-derived lymphoblasts, also consistent with these being DcpS-sensitive genes (Fig 3). It was noted that these genes exhibited different temporal responses to DAQ-DcpSi treatment: *PAQR8* was consistently down-regulated after 4 hours whereas the *DPM3* response required significantly longer treatment time with DAQ-DcpSi.

### Effect of DcpS inhibition on *SMN2* and *SMNΔ7* transcripts

**Fig 4:**
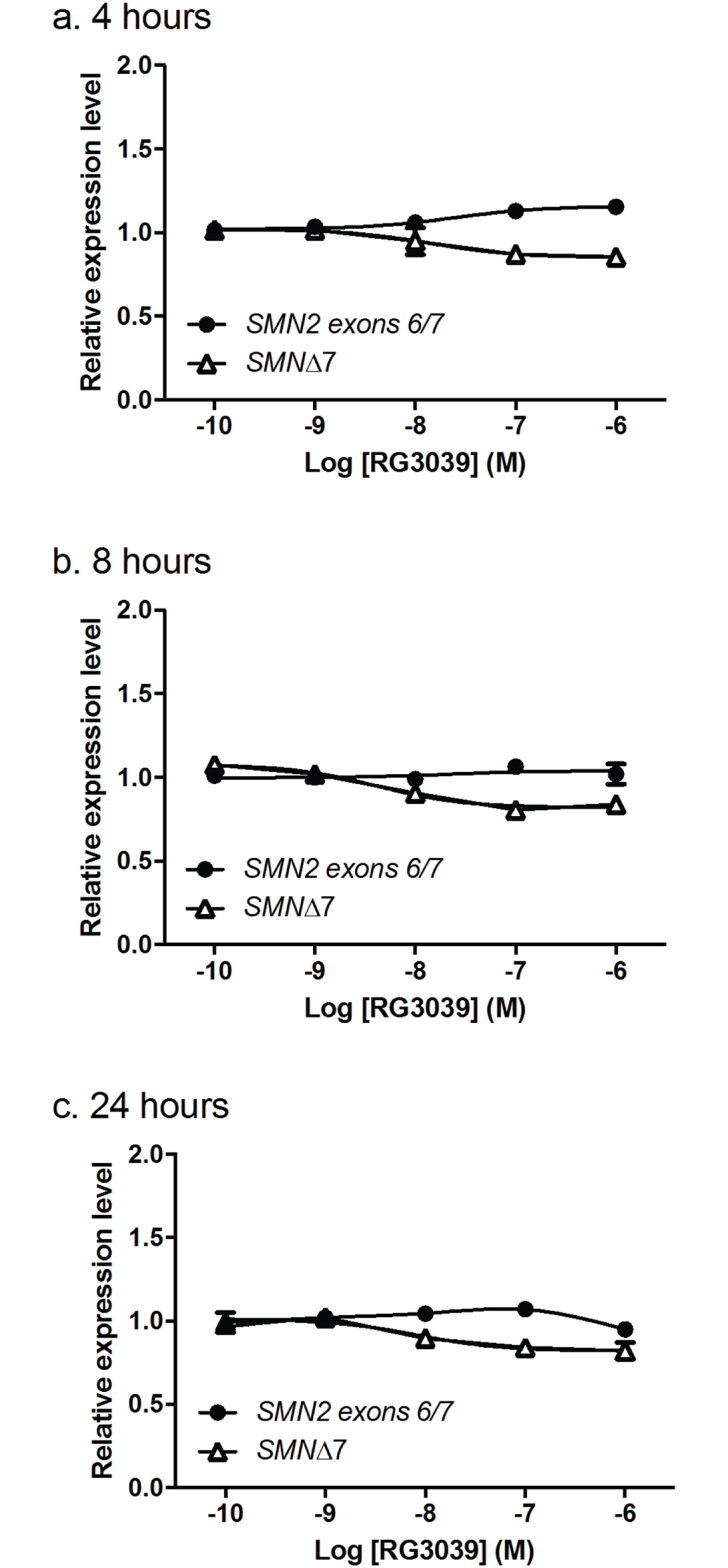
Effect of RG3039 on *SMN* transcripts. HEK-293T cells were treated with the indicated concentrations of RG3039 for various periods of time (4, 8 or 24hr) before the levels of *SMN* transcripts were measured using ddPCR. All data shown as mean ± s.e.m. and where not shown error bars are within the size of the symbols.

**Fig 5:**
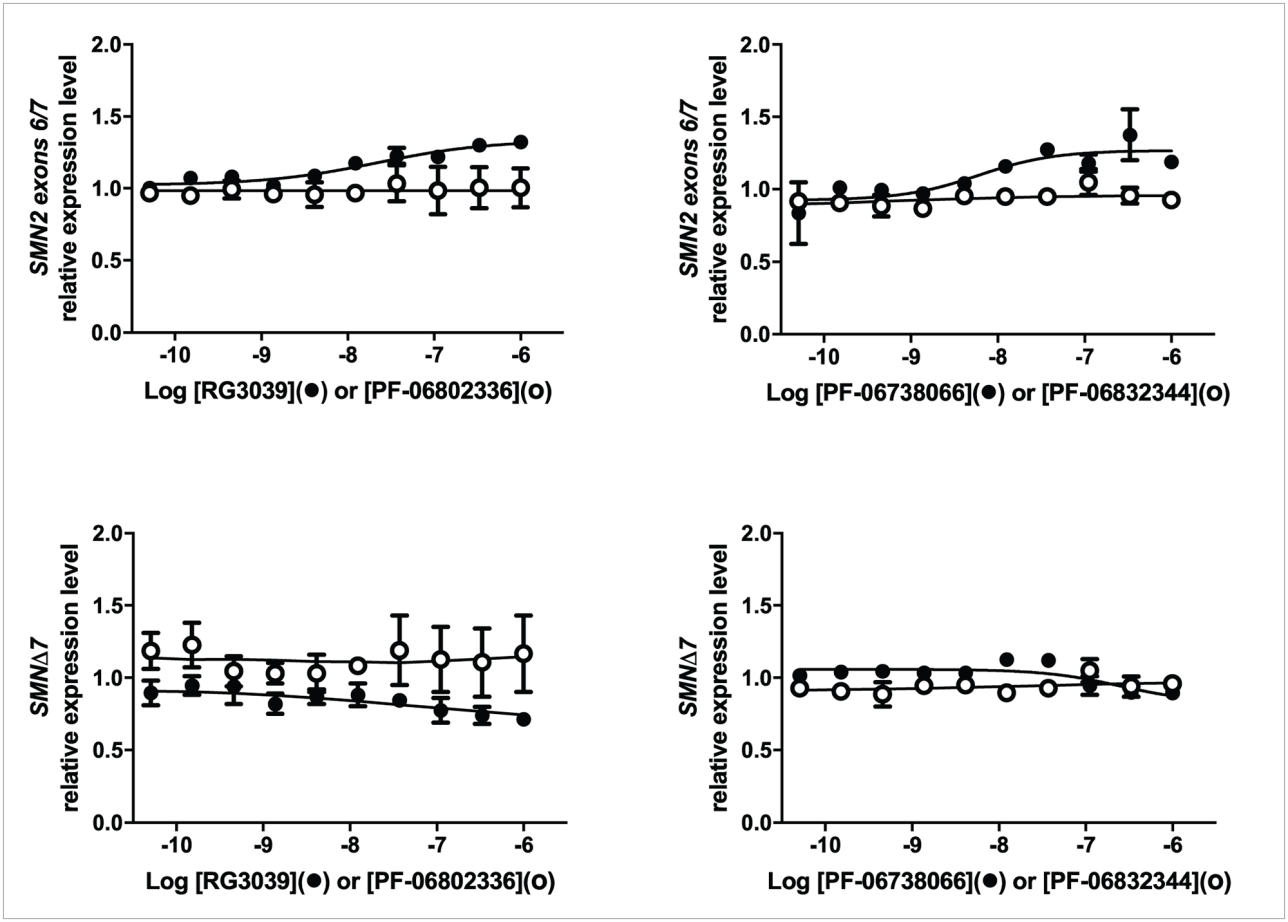
Effect of active DAQ-DcpSi and their 7-methyl analogues on *SMN* transcripts over time. SMA-derived lymphoblasts were treated with active DAQ-DcpSi (RG3039, PF-06738066) or their 7-methyl analogues (PF-06802336 and PF-06832344 respectively) and the levels of *SMN* transcripts measured using ddPCR. All data shown as mean ± s.e.m. and where not shown error bars are within the size of the symbols.

We utilized ddPCR to study the effect of RG3039 on *SMN* transcripts in a variety of different cell types—human peripheral blood mononuclear cells (PBMC), HEK283T, HelaS3, SMA lymphoblasts, SMA iPS-derived neural progenitor cells, and motor neurons. Typically (Fig 4), HEK293T cells responded with a small (~20%) increase in *SMN2* transcript after 4h of treatment with RG3039 which was much reduced or absent at later time points (Fig 4B and 4C): similar changes in *SMN2* were observed in HelaS3 and SMA lymphoblasts but no *SMN2* response was seen in any experiment conducted in PBMC or motor neurons. A small but sustained decrease in *SMNΔ7* transcripts was observed in all cells except PBMC. There were no changes in *SMN1* mRNA in response to treatment in any experiment in any cell type. We then went on to compare the effects of active DAQ-DcpSi (RG3039 and PF-06738066) with their 7-methyl inactive analogues (PF-06802336 and PF-06832344, respectively) and determined that the modest changes in *SMN* transcripts only occurred in response to active DAQ-DcpSi, but not 7-methyl analogues, consistent with these effects being a consequence of DcpS inhibition (Fig 5). In the particular example experiment illustrated in Fig 5 using SMA lymphoblasts, there was no decrease in *SMNΔ7* transcripts in response to RG3039, suggesting that this could be a cell-type specific response. However, in other experiments also performed in SMA lymphoblasts, as well as those done in SMA fibroblasts, a decrease in *SMNΔ7* transcripts was seen, so the lack of response is the result of experiment-to-experiment variation rather than a difference from one cell-type to another. No increase in *SMNΔ7* transcripts in response to DAQ-DcpSi was ever observed in any cell type *in vitro*.

### Effect of DAQ-DcpSi on SMN protein in SMA cells

**Fig 6:**
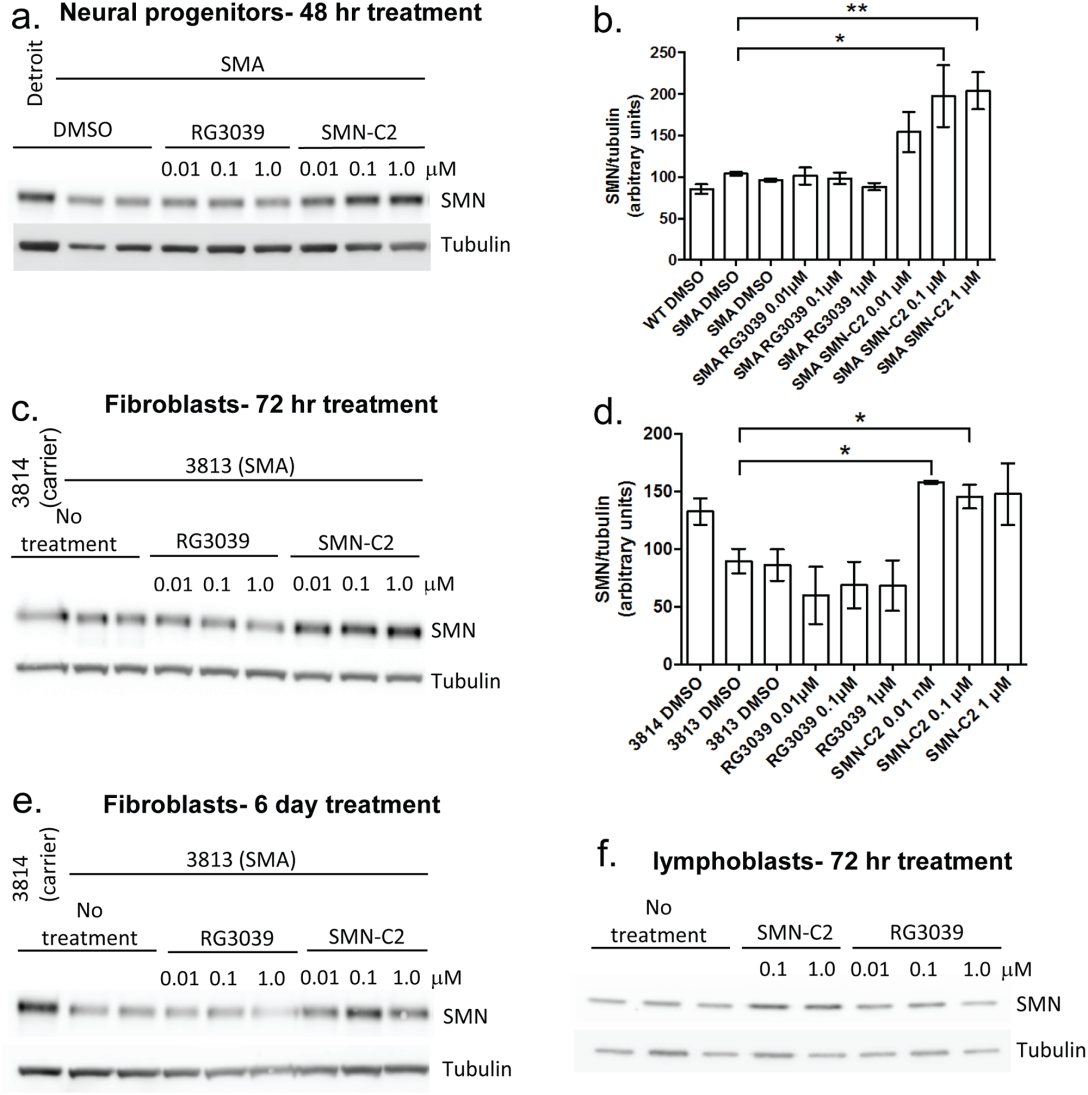
Effect of RG3039 on cellular SMN protein levels determined by Western blot. A. Neural progenitor cells treated for 48 hours (example) and B. quantification of data from 4 independent experiments; C. Fibroblasts treated for 72 hours (example) and D. quantification of data from 3 independent experiments; E. Fibroblasts treated for 6 days (example); F. SMA lymphoblasts treated for 72 hours (example). DMSO final concentration for lymphoblast cells was 0.1% and 0.2% for neural progenitors and fibroblast cell lines. All aggregate data is expressed as mean ± s.e.m. and statistical significance vs. SMA DMSO (B.) or 3813 DMSO controls (D) was assessed using Student’s *t*-test: *P*<0.05 (*), *P*<0.01(**) or otherwise non-significant where not indicated.

The effect of RG3039 on SMN protein was tested on a number of different SMA cell types under varying conditions (Fig 6). To validate our ability to detect the increase in SMN protein we included the compound SMN-C2, a *SMN2* splice-corrector [23], as a control and were able to clearly and reproducibly observe a positive SMN response to this compound in SMA fibroblasts, SMA lymphoblasts, and SMA iPS-derived neural progenitor cells (Fig 6). However, in no experimental condition tested were we able to detect an increase in SMN protein induced by RG3039 or any other DAQ-DcpSi after periods of incubation ranging from 24 hours to 7 days.

### DAQ-DcpSi effects in reporter gene assays are not specific to the *SMN2* promoter

**Fig 7:**
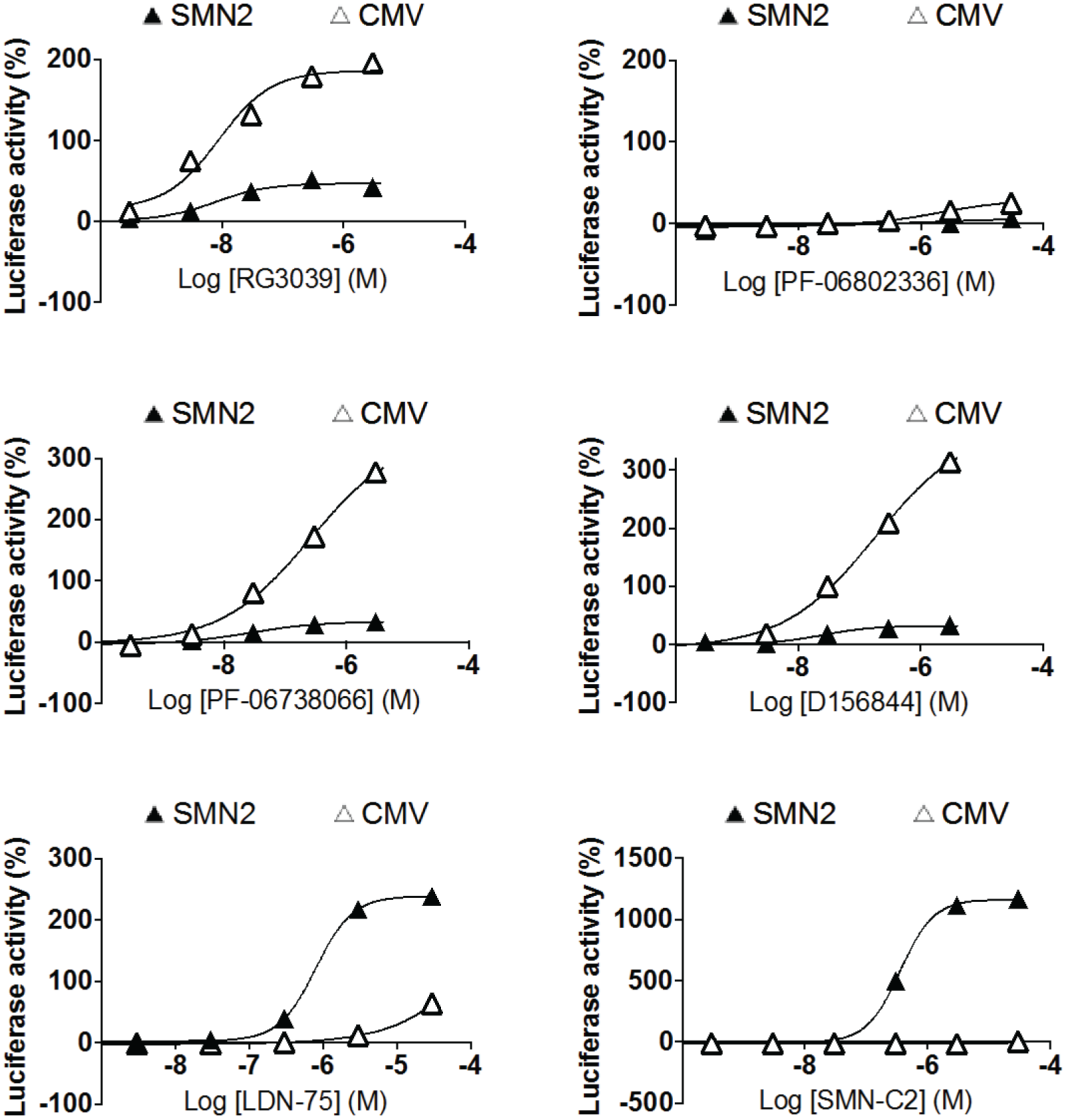
Effect of DAQ-DcpSi on luciferase activity driven by either the SMN2 promoter and SMN splicing cassette (filled triangles) or CMV promoter (open triangles). All data shown as mean ± range of duplicate determinations for a representative experiment of two performed which gave very similar results. Where not shown, error bars are within the size of the symbols.

A number of DAQ-DcpSi and their inactive 7-methyl analogues were profiled in a reporter assay described previously in which firefly luciferase is driven by the *SMN2* promoter and regulated by an *SMN2* splicing cassette [14]. This construct is present in an Epstein Barr virus vector maintained episomally in a stable cell line. This assay system is sensitive to *SMN2* transcriptional activators, splice correctors and compounds that stabilize either SMN-luciferase fusion transcripts or protein. *Renilla* luciferase driven by the *CMV* promoter in the same construct is employed as a useful comparator. As expected, the positive controls LDN-75654 [15] and SMN-C2 [23] had a strong positive effect on *SMN2*-driven firefly luciferase activity and had either a much weaker (LDN-75654) or no effect (SMN-C2) on *CMV*-driven *Renilla* luciferase activity (Fig 7). All 3 DAQ-DcpSi tested (RG3039, PF-06738066, and D156844 [9]) had a strong effect on *Renilla* luciferase activity. In contrast, the effect of the DAQ-DcpSi compounds on *SMN2*-firefly luciferase was of a smaller magnitude than their effect on *Renilla* luciferase (maximal activation ~20–50% compared to ~150–300% effect) but did appear concentration-dependent. In addition there was no effect of the inactive 7-methyl DAQ analog, PF-06802336 on the expression of either *SMN2*-firefly luciferase or CMV renilla, suggesting that the effect is a consequence of DcpS inhibition. These data are consistent with previous findings in a similar assay system in which early members of the DAQ series were found to have activity in a number of reporter gene assays in addition to (and in some cases greater than) that driven by the *SMN2* promoter [6]. They suggest that the primary effect of the DAQ-DcpSi are either non-specific promoter activation, or that these apparent promoter effects are either downstream of (or epiphenomena resulting from) some other primary pharmacological effect. These possibilities are consistent with the observation that members of this chemical series also caused activation of thymidine kinase promoter-driven β-lactamase activity [6]. It should also be noted that transcriptional activation of the *SMN2* gene would be predicted to increase *SMN*Δ7 transcript levels; however, as described above, the small elevations in *SMN2* transcript that were observed in cells *in vitro* are accompanied by either decreased *SMN*Δ7 or no change (see Figs 4 and 5).

### RNA Seq analysis of DAQ-DcpSi effects in N2a neuroblastoma cell

**Fig 8:**
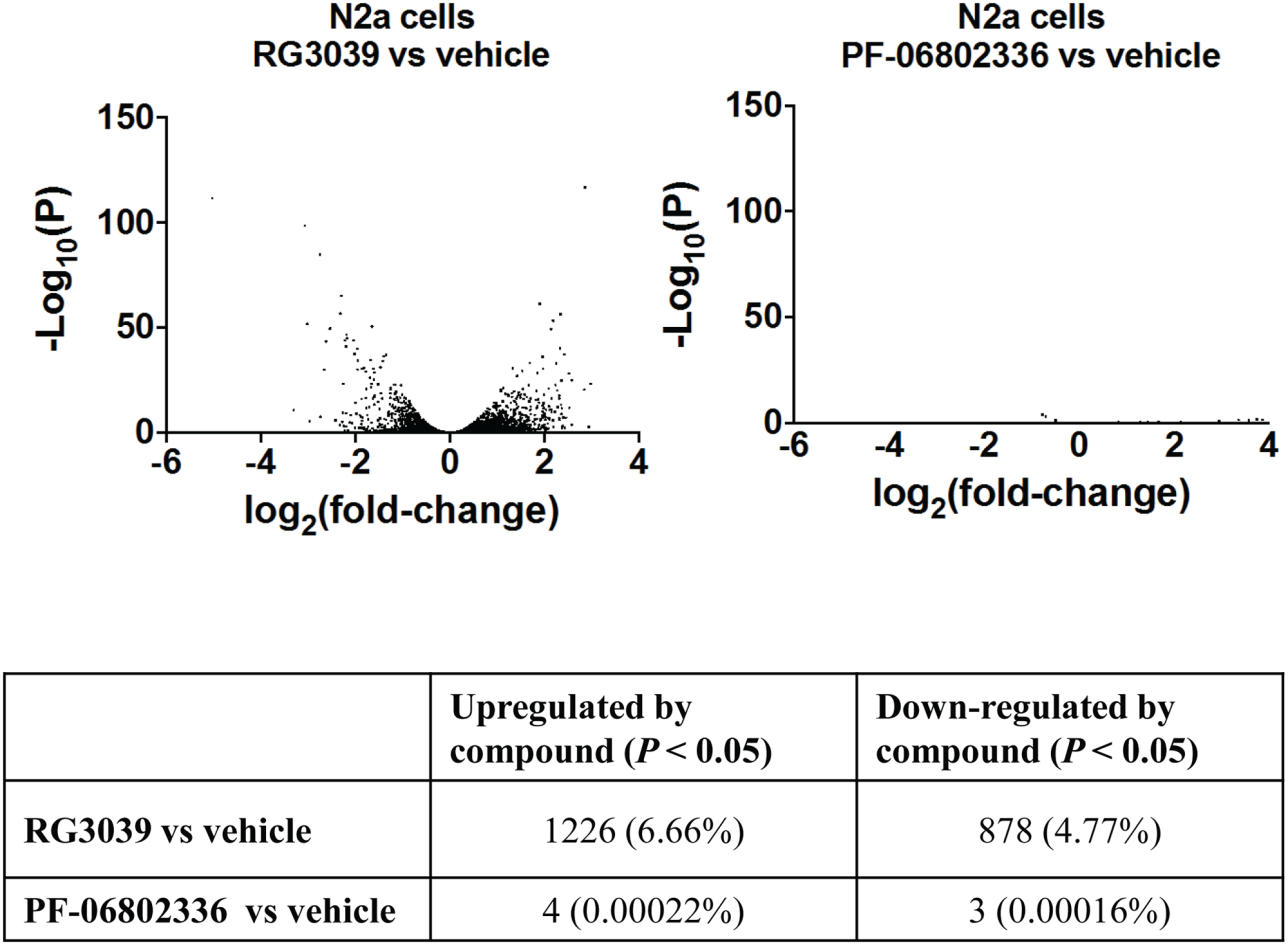
Volcano plots of differentially regulated genes detected by RNA-Seq analysis of N2a cells treated with RG3039 or its 7-methyl analog PF-06802336 with summary of number of genes significantly (*P*<0.05) changed. Plates of cells (in quadruplicate) were treated with 1 μM of either compound or DMSO (1 μM) for 24 hours before isolation of polyA RNA and RNA Seq analysis. In each case, the—Log10 of the corrected *P* value for the difference between compound- and DMSO-treated samples for each detectable gene was plotted vs the Log2 of the fold difference. The table summarizes the numbers of significantly (*P*<0.05) differentially-regulated genes irrespective of the fold change and as a percentage of the total numbers of genes detectable. A total of 18,409 genes were detectable.

In order to more broadly characterize the effects of DcpS inhibition on the transcriptome, N2a mouse neuroblastoma cells were treated for 24 hours with either 1 μM RG3039, 1 μM PF-06802336 or an equivalent concentration (1 μM) of DMSO vehicle. RNA-Seq analysis on a polyA RNA library prepared from these cells revealed 2,104 genes differentially expressed in RG3039-treated cells compared to DMSO-treated controls out of 18,409 detectable genes (*P* < 0.05). Of these, 1,225 genes (58.3%) were upregulated and 878 (41.7%) were down-regulated (Fig 8). It was noted that neither *Smn* nor *DcpS* itself were differentially expressed in response to RG3039 treatment by these significance criteria. In contrast, only 7 detectable genes were differentially regulated in PF-06802336-treated cells in comparison to DMSO controls. Four of these 7 genes were also differentially regulated by RG3039, and all 4 changed in the same direction with both compounds (all upregulated) indicating regulation by the chemotype independent of DcpS inhibition for this small set of genes (RNA Component of Mitochondrial RNA Processing Endoribonuclease (*Rmrp*); *predicted gene 15564* (*Gm15564*); *7SK Small Nuclear RNA* (*Rn7sk*); and *mmu-miR-6236*). After removal of these 4, a complete list of the genes differentially regulated by RG3039 in Neuro2a cells is provided in S4 Table. 1,489 (70.9%) of these genes changed by less than one Log_2_ units (2-fold) in either direction, and only 80 genes (3.8%) changed by more than 2 log_2_ units (4-fold) in either direction. Thus a substantial proportion (>11%) of the detectable genes in these cells are affected by RG3039 but the vast majority change only modestly. Ingenuity Pathway Analysis (IPA) indicated that the affected genes were distributed broadly, with no pathway(s) standing out as being a selective target for the action of the compound in providing therapeutic benefit in SMA. 53 pathways were significantly affected (*P* < 0.05) (S1 Fig). Of these, the great majority had approximately equal representation of genes that were up-regulated and those that were down-regulated, making it unclear what the likely overall effect on the pathway was likely to be. The only exceptions to this general pattern were pathways that had very few genes annotated (S1 Fig) which is likely a statistical anomaly. When viewed in terms of disease and biofunction annotations, differentially affected genes were also broadly distributed, also with approximately even distribution of directionality of change (S2 Fig). Thus no obvious hypotheses for therapeutic mechanism emerged from this RNA-Seq analysis. Consistent with our ddPCR findings in human cells reported above, in these mouse neuroblastoma cells treated with DAQ-DcpSi, *Paqr8* was decreased (log_2_ fold change 0.657, *P* = 0.00251), *Dpm3* was increased (log_2_ fold change 0.884, *P* = 0.000012), as was *Pigw* (log_2_ fold change 1.54, *P* = 1.89 x 10^−30^; S4 Table). Due to the implication of miRNA dysregulation in SMA and a role for DcpS in miRNA stability, we hypothesized that DAQ-DcpSi may regulate gene expression via changes in miRNA levels. However, RNA-Seq analysis on miRNA isolated from treated and untreated N2a cells found no miR species (out of approximately 700 detectable) that were differentially regulated by RG3039 but not its inactive 7-methyl analog PF-06802336. These data suggest either that DAQ-DcpSi do not regulate miRNA levels, or that any changes are too subtle to be detected using RNA-Seq methodology. The latter interpretation appears most likely based on a demonstrable effect of DAQ-DcpSi reported recently [24].

### DAQ-DcpSi therapeutic benefit in SMA mice is not the result of lysosomotropism and is accompanied by significant and substantial increases in *Smn* transcripts but no change in Smn protein

**Fig 9:**
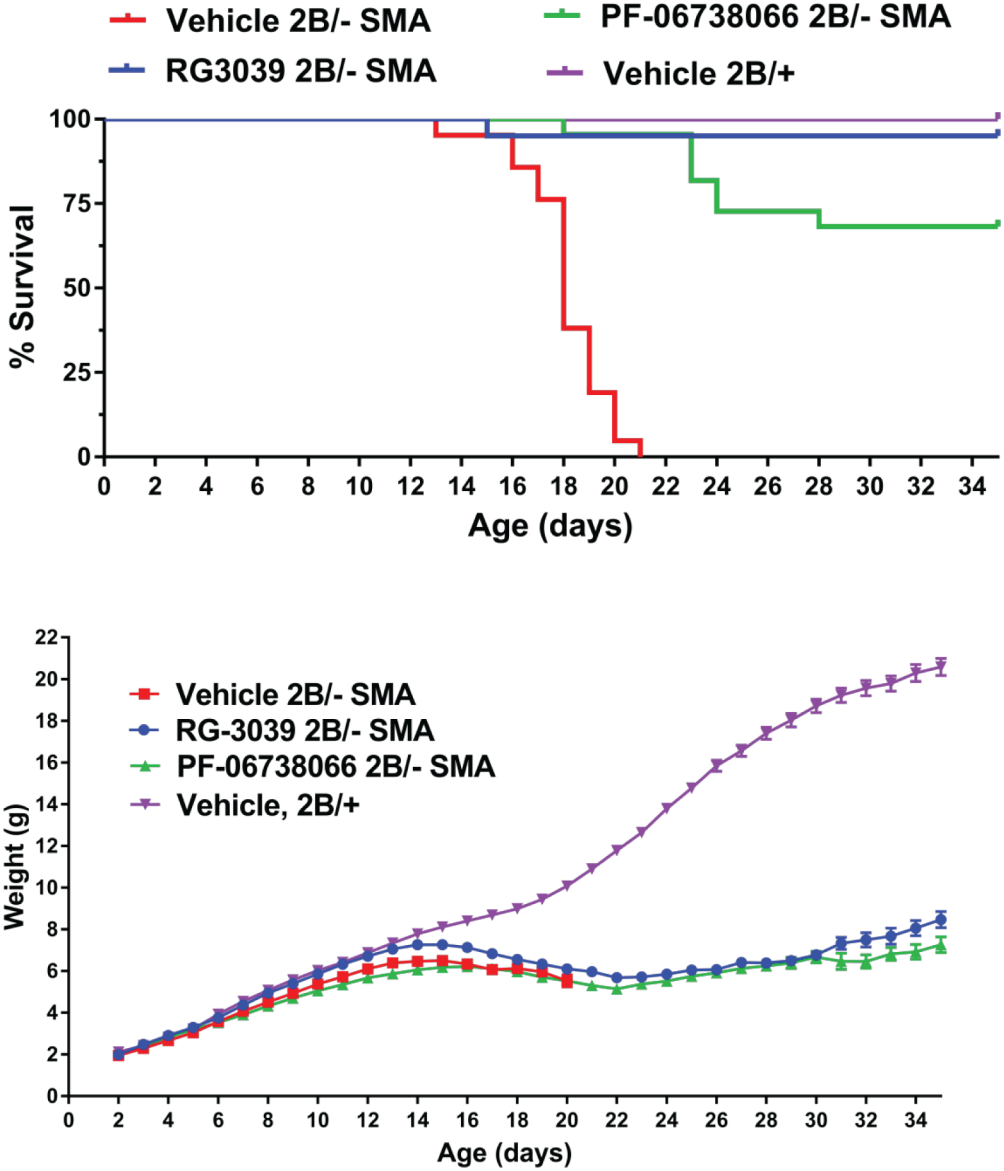
Effect of DAQ-DcpSi on survival and body weight of 2B/- SMA mice. RG3039 (6 mg/kg) or PF-06738066 (10mg/kg) were dosed b.i.d. via intraperitoneal injection using a dosing volume of 2.5 μl/g and controls received an equal volume of vehicle. Healthy 2B/+ littermate controls dosed with vehicle were included for comparison. Data presented represent combined sexes for RG3039 (*n* = 20); vehicle (*n* = 21), PF-06738066 (*n* = 21) and vehicle treated healthy 2B/+ littermates (*n* = 24). All data shown as mean ± s.e.m. and where not shown error bars are within the size of the symbols. RG3039 and PF-06738066 had a significant beneficial effect on survival of SMA mice compared to vehicle SMA mice (*P* < 0.0001 by either Mantel-Cox or Gehan-Breslow-Wilcoxon tests). RG3039 had a slightly greater effect on survival than PF-06738066 (*P* < 0.05 by either test).

The 2 DAQ-DCpSi compounds that have been demonstrated to have beneficial effects in SMA mice *in vivo* (RG3039 and D156844) are both highly lipophilic and dibasic [7–9]. Compounds with these physicochemical properties accumulate in tissues as the result of partitioning into membranes and sequestration within the lysosomal compartment due to the formation of protonated membrane-impermeable species at low pH [25]. As so-called lysosomotropes have been found to be neuroprotective [26], we wanted to determine if the DAQ-DcpS inhibitors could show therapeutic benefit for SMA mice without accumulating to the lysosome. PF-06738066, an analog of RG3039 that we have previously shown to be a potent DAQ-DcpSi but non-lysosomotrope [12], was tested in the 2B/- SMA mouse model. Preliminary pharmacokinetic (PK) profiling of PF-06738066 [30 mg/kg i.p.] (S3 Fig) was performed in adult FVB/N (wild type, Smn^+/+^) mice and showed moderate brain penetration—free drug levels in brain and CSF were approximately 2.5-fold lower than in plasma. This profiling was extended to 2B/- SMA mice and 2B/+ littermate controls at P13 using a dose of 10 mg/kg i.p. (S4 Fig). It was determined that drug exposures were somewhat higher in SMA pups compared to their healthy littermates (S4B Fig), and that pups have higher CNS drug exposures as a function of dose compared to adult mice. Free drug levels in 2B/- SMA pup brains were 3–4 fold lower than in plasma. These PK data indicated that 12 hr following a 10 mg/kg dose free drug levels in brain were approximately 7 nM which we expect to result in significant DcpS inhibition if given b.i.d. (S4 Fig). These CNS distribution characteristics of PF-06738066 are in marked contrast to those of RG3039 that (as expected from its physicochemical characteristics) significantly accumulates in all tissues compared to plasma [7]. We then studied the effects of dosing PF-06738066 (10mg/kg b.i.d. i.p.) or RG3039 (6 mg/kg b.i.d. i.p.) to 2B/- SMA mice on survival, motor function, and *Smn* transcripts in muscle and spinal cord. The study was powered to detect a survival benefit and the dosing regimens were selected to maximize the likelihood of achieving a maximal degree of DcpS inhibition in CNS tissues over the dosing cycle. Both PF-06738066 and RG3039 had a statistically significant beneficial effect on survival of SMA mice compared to vehicle-treated SMA mice (*P* < 0.0001 by either Mantel-Cox or Gehan-Breslow-Wilcoxon tests) and RG3039 had a slightly greater effect on survival than PF-06738066 (*P* < 0.05 by either test) (Fig 9). Males tended to survive longer than females in all groups but this was statistically significant only within the vehicle-treated SMA mice. As previously reported for RG3039, animals treated with either DAQ-DcpSi compound had a decline in weight around the time of weaning and then gained weight thereafter but were nevertheless always significantly lower in their body weight than healthy 2B/+ control animals (Fig 9).

**Fig 10:**
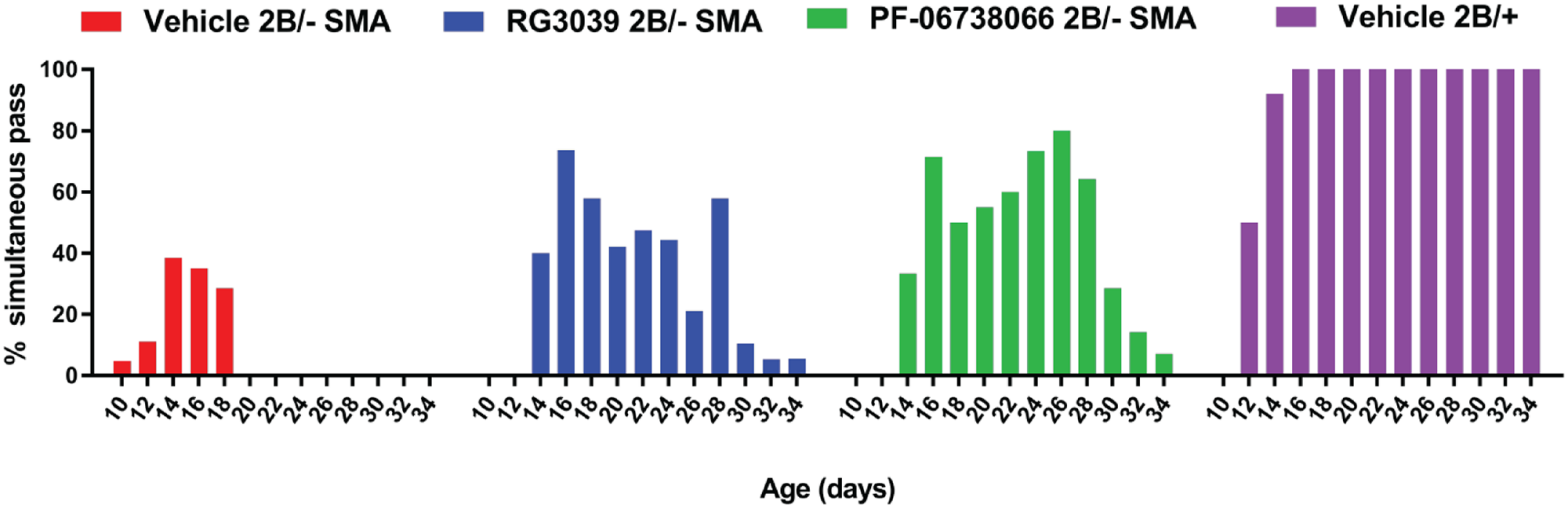
Effect of DAQ-DcpSi on the performance of 2B/- SMA mice in a combined 55° negative geotaxis/ climb test. Data presented represent combined sexes for RG3039 (*n* = 20); vehicle (*n* = 21), PF-06738066 (*n* = 21) and vehicle treated healthy 2B/+ littermates (*n* = 24). The percent of mice able to pass the 55° negative geotaxis and climb tests simultaneously is shown. At P16, while a predominant number of vehicle treated SMA were alive, PF-06738066 or RG3039 showed improvement over vehicle SMA mice that did not reach statistical significance (Fisher’s exact test, *P*-value = 0.0708; 43% power to detect a benefit amongst SMA groups).

To assess motor function and endurance, mice were monitored every other day from P10-P34 for their ability to simultaneously pass a 55° negative geotaxis and climb test (Fig 10). By P16, 100% of the vehicle-treated control 2B/+ mice were able to simultaneously pass the combined assay, while at the same age, less than 40% of SMA mice (combined sexes) treated with vehicle that were still alive were able to pass. In contrast, those SMA mice that received either PF-06738066 or RG3039 had a simultaneous pass rate of about 78%- almost a 40% improvement in their ability to pass the combined assay, but this did not reach statistical significance (Fisher’s exact test, *P*-value = 0.0708). This assay amongst the SMA groups had a 43% power to detect a significant change because of the small n-value available at P16 and the mixed responses among the SMA groups (compared to >99% power between control and SMA because of the 100% pass rate in control mice). Post-dosing SMA drug-treated mice demonstrated a gradual decline in strength and endurance. It is unknown whether this loss would have occurred if drug dosing had been maintained beyond P20. In the second part of this study, plasma drug levels were measured 12 hours following the last dose (T_min_) on P16 and after conversion for plasma protein binding these equated to plasma free drug concentrations of 4.24 ± 1.13 nM RG3039 (n = 13) and 184 ± 76 nM PF-06738066 (n = 18) respectively with no obvious sex differences apparent on subgroup analysis, confirming that the achieved drug levels equaled or exceeded the targeted range for each drug based on the preliminary PK analysis in both adult wild type and younger SMA pups.

### Smn transcript and protein analysis on tissues from DAQ-DcpSi-treated mice by ddPCR

**Fig 11:**
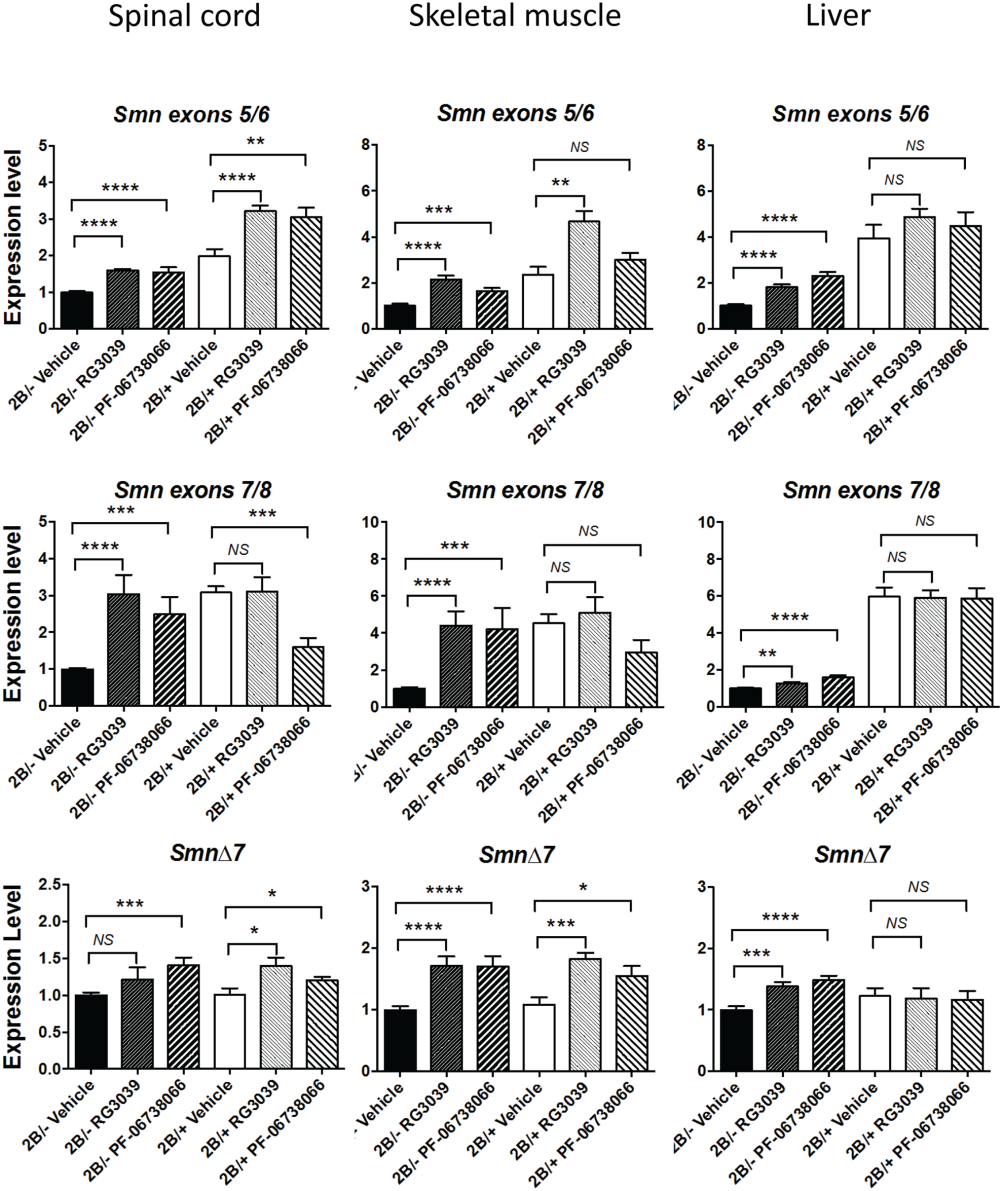
Effect of DAQ-DcpSi treatment on *Smn* transcript levels in tissues from 2B/- SMA mice and healthy 2B/+ littermate controls. Animals were dosed with either vehicle, RG3039 (6 mg/kg) or PF-06738066 (10 mg/kg) BID via intraperitoneal injection from P4-P16 and were sacrificed 12 hours following the last dose for collection of tissues. RNA was prepared and analyzed using ddPCR as described in materials and methods. All gene expression was normalized to *PSMD14* expression and expressed relative to that in vehicle-treated 2B/- mice. All data shown as mean ± s.e.m. Numbers of animals in each data set were: 2B/- Vehicle (22); 2B/- RG3039(9); 2B/- PF-06738066 (13); 2B/+ Vehicle (7); 2B/+ RG3039(9); 2B/+ PF-06738066 (10). Significance using Student’s *t*-test: *P*<0.05 (*), *P*<0.01(**), *P*<0.001(***), *P*<0.0001(****).

**Fig 12:**
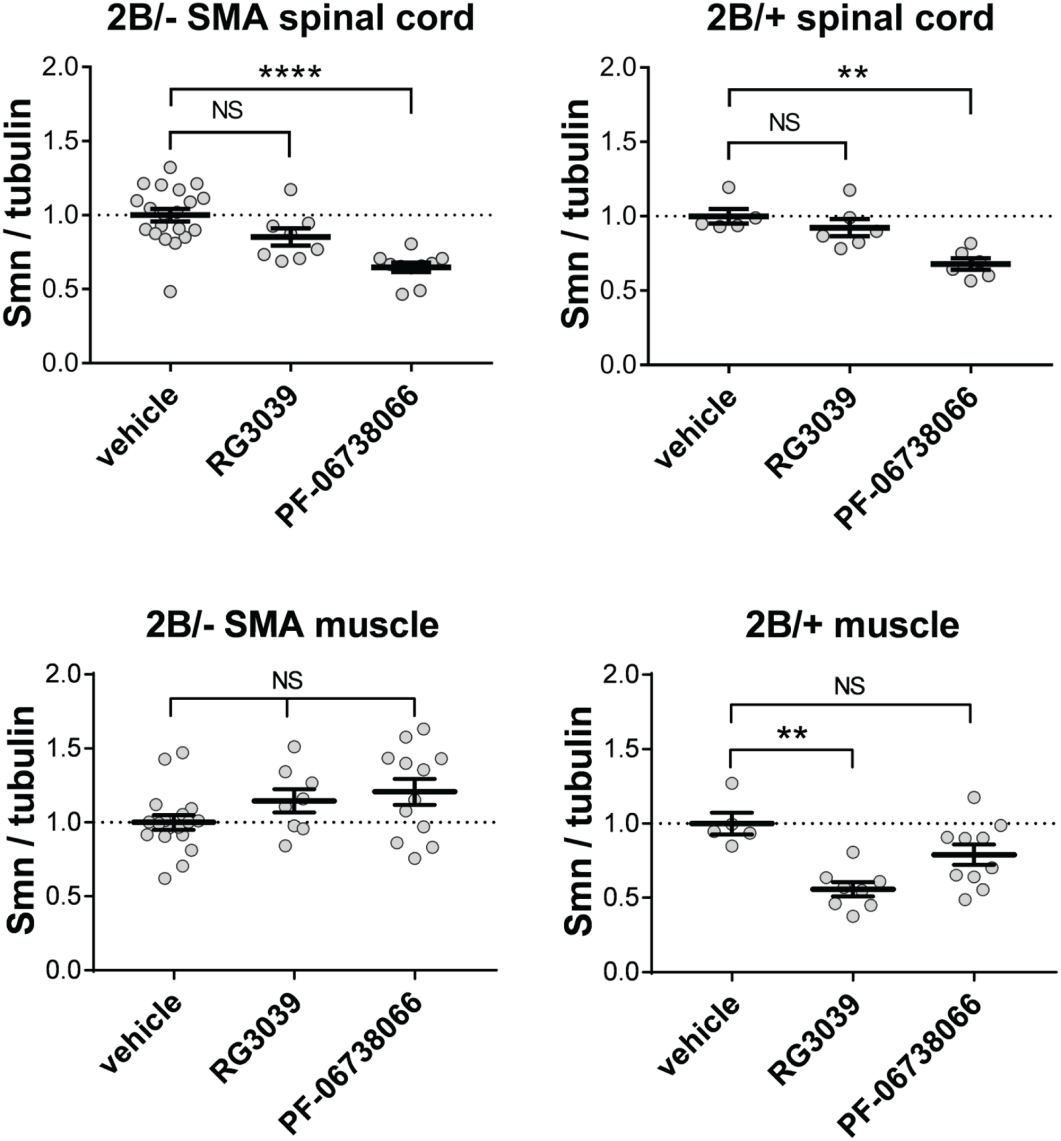
Smn protein levels in spinal cord and muscle tissue from 2B/- SMA mice or 2B/+ healthy littermates treated with either vehicle, RG3039, or PF-06738066. Tissues were collected as described in Materials and Methods. Data is expressed as mean s.e.m. and statistical significance was evaluated using One-way ANOVA and Dunnett's multiple comparisons test. Tukey post-hoc tests were utilized to determine significance among the groups. *P*<0.05 (*), *P*<0.01(**), *P*<0.001(***), *P*<0.0001(****).

As expected, 2B/- SMA mice had lower levels of exon 5/6 and exon 7/8 containing *Smn* transcripts in all tissues compared to 2B/+ controls (Fig 11). Levels of *Smn*Δ7 transcripts (defined by contiguous exons 6 and 8) were identical between the 2B/- SMA mice and their healthy 2B/+ littermates, presumably because each bears a single copy of the 2B allele that is the source of this transcript. 2B/+ mice were used for these analyses as they produce ~65% Smn protein, are phenotypically normal and were littermate controls to the SMA mice analyzed from the breeding scheme that was utilized. In contrast to the modest effects observed in cells treated *in vitro*, both RG3039 and PF-06738066 caused significant increases in *Smn* transcripts in all tissues of 2B/- SMA mice with the exception that *Smn*Δ7 trended to increase in spinal cord of RG3039-treated 2B/- mice but this apparent effect did not achieve statistical significance. These increases were observed in exons 5/6-containing *Smn*, exons 7/8-containing *Smn*, and *Smn*Δ7 transcripts. It is notable that in spinal cord and muscle, exon 7-containing transcripts were fully normalized to the levels observed in healthy 2B/+ mice but the increase in liver achieved only partial normalization. While 2B/+ control mice similarly responded to DAQ-DcpSi with an increase in exons 5/6-containing *Smn* and *Smn*Δ7, there was no increase in exons 7/8-containing *Smn* transcripts. In spinal cord PF-06738066 treatment actually caused a significant decrease in these *Smn* exons 7/8 and a similar trend was apparent in skeletal muscle but did not achieve statistical significance. These data suggest that the *Smn* response to DAQ-DcpSi treatment *in vivo* is more complex than a simple increase in expression as might be expected by increased promoter activity. In addition RG3039 caused no statistically significant change in Smn protein levels in spinal cord or muscle tissues from 2B/- SMA whereas PF-06738066 caused a significant decrease in spinal cord (*P*< 0.0001) and a small but significant increase in muscle (*P*< 0.05) from these mice (Fig 12, and S5 Fig for representative blots). Thus the observed changes in *Smn* transcripts were not associated with elevation of Smn protein and the mechanism(s) of therapeutic benefit of these compounds remain cryptic but not due simply to overall increases in Smn protein.

### RNA-Seq analysis of gene expression changes induced by RG3039 in SMA mice

**Fig 13:**
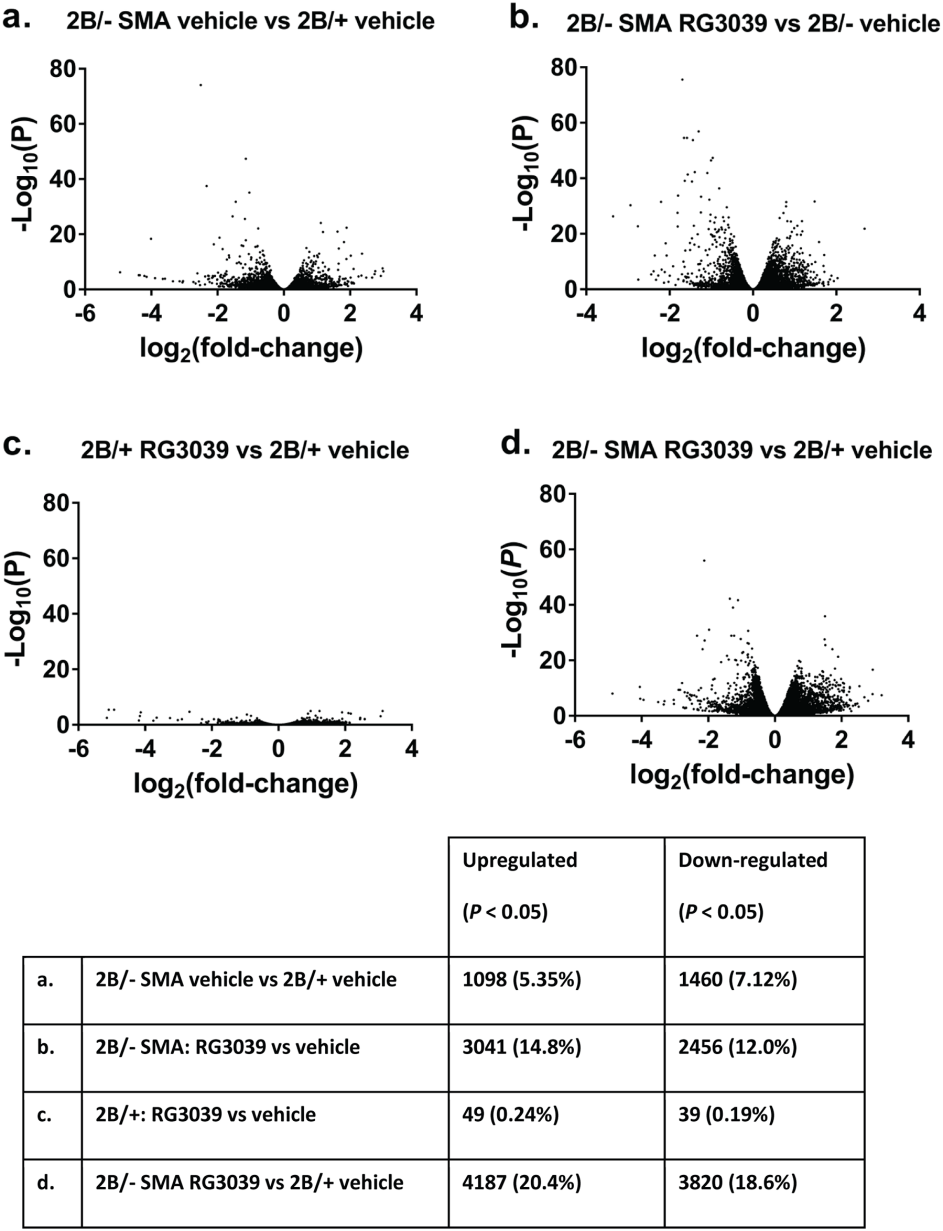
Volcano plots of differentially regulated genes detected by RNA-Seq analysis of spinal cord tissues from 2B/- SMA mice or 2B/+ healthy littermates treated with RG3039 or vehicle. The table (E.) summarizes the number of significantly (*P*<0.05) differentially-regulated genes irrespective of the fold change for the various comparisons and as a percentage of the total numbers of genes detectable. Upregulated is defined such that the first condition in the comparison caused upregulation relative to the second e.g. 3,041 genes were upregulated by RG3039 treatment of SMA mice in comparison to vehicle-treated SMA mice. For all the comparisons the total number of detectable genes was between 20,514 and 20,517.

Following the observations described above, we went on to more completely characterize the pattern of changes across the transcriptome by performing RNA-Seq analysis on polyA RNA libraries from spinal cord tissues from the *in vivo* experiment described above. Spinal cord tissue from 2B/- SMA and 2B/+ control mice treated with vehicle or RG3039 (6 mice per condition) was used for RNA-Seq analysis. Comparison of vehicle-treated animals (2B/- SMA vs 2B/+ control) was used to identify those genes whose expression was altered in diseased vs healthy animals. Two of the 6 samples from vehicle-treated 2B/+ mice had markedly higher expression of skeletal muscle specific genes which we interpreted as likely contamination of these spinal cord tissues with skeletal muscle during the dissection process and therefore these individual animals were excluded from the analysis. We found 2,558 genes were differentially expressed in spinal cord from 2B/- SMA mice compared to healthy 2B/+ littermates with a slight preponderance of genes that were lower in 2B/- SMA vs 2B/+ (7.12% vs 5.35% upregulated) (Fig 13A and 13E and S5 Table for underlying data). Transcripts previously identified as being dysregulated in SMA tissues in general as well as in 2B/- SMA mice were also confirmed in this data set [*eg*. *Cdkn1a (cyclin-dependent kinase inhibitor 1A (P21)*, *Pmaip*1, (phorbol-12-myristate-13-acetate-induced protein 1) and *Fas* (TNF receptor superfamily member 6)] [27]. The top 20 up and down differentially expressed transcripts are given in the S6 Table. As expected, vehicle-treated 2B/- mice had 2.33-fold lower *Smn* transcript levels compared to vehicle-treated 2B/+ mice (Log_2_ fold change = -1.224, *P* = 1.72 x 10^−16^). Strikingly, dosing of 2B/- SMA mice with RG3039 resulted in significantly (*P* < 0.05) altered expression of 5,497 genes compared to vehicle-treated 2B/- mice SMA mice (approximately 26.8% of the measurable genes), which was a significantly greater proportion than the 11.4% of measurable genes found to be modulated in the N2a cell RNA-Seq analysis (Fig 13B & 13E. and S7 Table for underlying data). The top 20 up and down differentially expressed transcripts are found in the S8 Table. RG3039 treatment did not appear to increase *Smn* transcripts as detected by RNA-Seq, possibly reflecting the lower sensitivity of RNA-Seq compared to ddPCR in ability to detect changes in low-abundance transcripts.

In marked contrast to the data in 2B/- SMA animals, less than 0.5% of detectable genes were modulated by RG3039 in 2B/+ control mice (Fig 13C and 13E and S9 Table for underlying data). Based on these findings we hypothesized that the therapeutic benefit of RG3039 could be either due to, or at least reflected by, the modulation of a subset of genes that were affected in one direction by disease and in the opposite direction by RG3039 treatment of SMA mice, returning the tissue to a more “healthy” gene expression profile. To investigate this hypothesis we compared differential expression between RG3039-treated 2B/- mice and vehicle-treated 2B/+ controls (Fig 13D). Contrary to our hypothesis, which would have predicted fewer differential genes, we found a much greater number of genes were different in this comparison than between vehicle treated 2B/- and vehicle-treated 2B/+ (compare Fig 13A and 13D), indicating that compound treatment actually made 2B/- tissue less “similar” to healthy tissue than the untreated 2B/- tissue (see S10 Table for underlying data). 591 genes were affected in opposite directions by disease and by compound treatment (see S11 Table for list of genes). Pathway analysis performed in IPA software indicated a significant number of pluripotency and cancer-related genes in this list. As a final hypothesis we also looked at the expression of transcripts such as *ZPR1* whose overexpression has been shown to increase the number of SMN containing gems in SMA patient cells [28]. The idea being that Smn levels may not change, but subcellular distribution of Smn may, which we previously reported within SMA motor neurons of 2B/- SMA mice treated with RG3039 [8]. We found no difference in *ZPR1* expression in whole spinal cord by RNA-seq in SMA vs. Control 2B+ vehicle treated mice or amongst vehicle and RG3039 treated SMA mice (S5–S8 Tables).

## Discussion

Based on the mode of discovery of the DAQ-DcpSi in a screen utilizing an *SMN2* promoter-driven reporter assay and reported effects on *SMN2* transcripts, SMN protein levels and nuclear gem counts, this class of compound have been assumed to exert their therapeutic effects in SMA mice via elevation of SMN consequent to *SMN2* promoter induction. However, close examination of the published evidence indicates that the data underpinning this claim is equivocal at best. Firstly, the original hits in the quinazoline series were also active in a minimal thymidine kinase promoter-driven reporter assay, and were actually more potent in this context than they were in the *SMN2* promoter assay [6]. The reporter gene assay data reported in the current work replicates this finding in yet another unrelated (CMV) promoter-driven reporter assay and indicates that DAQ-DcpS are either not simple transcriptional activators of the *SMN2* gene, or if so, they are extremely non-selective transcriptional activators of a number of promoters or act via stabilization of transcripts. Secondly, although we have confirmed the observation of others that DAQ-DcpSi increases the ratio of full length *SMN2* transcript to *SMN*Δ7 transcript in cells *in vitro*, we show that the changes in either individual transcript are extremely modest and in the case of full length *SMN2*, are generally not sustained beyond 4 hours. It should be noted that expression of these changes in terms of the ratio between full length *SMN2* and *SMN*Δ7 transcripts as used by some authors is likely to exaggerate the magnitude of the potential beneficial effect, the significance of which is questionable especially given the observation that introduction of a *SMNΔ7* transgene actually improves the phenotype of SMA mice [29]. The modest effects of DAQ-DcpSi on full length *SMN2* transcripts that we have demonstrated *in vitro* are possible to detect using the exquisitely sensitive ddPCR methodology that we have used, but are unlikely to be reliably measured using more traditional qPCR techniques, possibly accounting for the inconsistency of the literature in this regard. ddPCR is an extremely powerful single molecule counting technique, is capable of quantitating minute quantities of low abundance transcripts, and is essentially linear in response (as opposed to the logarithmic responsiveness of traditional qPCR). This makes ddPCR uniquely suitable for detecting small compound effects on transcript levels. Another crucial aspect of the methodology is the choice of reference gene with which to normalize expression data. As illustrated by our RNA-Seq data, many genes are affected by DAQ-DcpSi treatment, including some commonly used as “housekeeping” reference genes. In addition, it is clear that ddPCR is more sensitive than RNA-Seq for detecting changes in low-abundance transcripts. Therefore it is vital that carefully controlled preliminary experiments are performed to select an appropriate reference gene to avoid exaggerating, underestimating, or even negating the effects of compounds such as DAQ-DcpSi treatment which clearly have very broad effects across the transcriptome. Using this approach we selected PSMD14 as an appropriate reference gene because it was reliably unaffected by DAQ-DcpSi treatment *in vitro*. Using these methodological approaches we are confident that the small changes that we observed in *SMN* transcripts are genuine, but seem very unlikely to be significant to the therapeutic mechanism of action of DAQ-DcpSi. The small effects of DAQ-DcpSi on *SMN* transcripts we describe in our *in vitro* experiments are in marked contrast to the robust changes caused by small molecule *SMN2* splice correctors reported by others [23], which reduce the *SMN*Δ7 transcript to undetectable levels and double full length *SMN2* levels. Increases in SMN protein in SMA fibroblasts *in vitro* in response to DAQ-DcpSi have been claimed by others but not quantitated. The contrast with the data we report in this paper could arguably be related to a different incubation period employed by other investigators (5 days) compared to our own studies. However this seems unlikely as we employed incubation periods both shorter and longer than this (up to 6 days) in our studies.

Lipophilic and basic DAQ-DcpSi accumulate within the lysosomal compartment due to the combination of being able to freely enter this organelle by virtue of high membrane permeability but then becoming positively charged by protonation at low pH, reducing membrane permeability and causing entrapment. This property (known as lysosomotropism) is likely responsible at least in part for the high degree of tissue accumulation of these compounds in tissues [8]. As lysosomotropes have previously been reported to be neuroprotective we sought to develop DAQ-DcpSi that had reduced lysosomotropism to test whether they retained their ability to improve disease phenotype in SMA mice [12]. In the current study our characterization of these mice was limited to survival and performance in a motor function task, but previous studies [7, 8] have shown improvement in electrophysiological parameters and neuromuscular junction architecture by RG3039, which are highly relevant to the pathology of SMA in humans. Therefore measurements of survival and motor function in these mice are an appropriate surrogate endpoint for neuromuscular function in this mouse model. The finding that a non-lysosomotropic DAQ-DcpSi has a beneficial effect similar to RG3039 excludes lysosomotropism as a potential therapeutic mechanism.

A striking finding in the present study was that despite our inability to demonstrate robust changes in *SMN* transcripts in cultured cells *in vitro*, DAQ-DcpSi treatment of SMA mice caused marked upregulation of exon 7-containing transcripts in spinal cord and skeletal muscle tissues, and to a lesser extent in liver. In these tissues, exon 5 & 6 containing and *Smn*Δ7 transcripts (in contrast to our *in vitro* findings) also generally increased. At present it is not possible to distinguish whether the difference between our *in vitro* and *in vivo* findings is the result of a difference in the length of exposure to the drug (24 hours *in vitro* vs. 12 days *in vivo*), cell type differences, or non-cell autonomous effects *in vivo* which are not mimicked *in vitro*.

In previous studies performed in SMA mouse models treated with therapeutically beneficial doses of DAQ-DcpSi, *SMN* full length transcripts have been reported to either increase modestly [7], or not to change [8, 9]. In contrast to this, and to our findings *in vitro*, we found relatively robust *in vivo* increases in *Smn* transcripts containing contiguous exons 7 and 8, as well as significant increases in those containing contiguous exons 5 and 6, and Δ7 transcripts (contiguous exons 6 and 8).

No statistically significant increases in SMN protein have been reported in SMA mouse models treated with DAQ-DcpSi under conditions where the compounds have been demonstrated to have a survival and functional benefit [7–9]. Overall our own data are consistent with DAQ-DcpSi causing no change in tissue levels of SMN protein. However it should be noted that nuclear gems do appear to increase both in SMA cells *in vitro* and in tissues from SMA mice dosed *in vivo* [8]. Indeed the intriguing suggestion has been made that this results from redistribution of SMN into the nucleus caused by DAQ-DcpSi, suggesting that although an elevation in total cellular SMN protein levels is widely assumed to result in an increase in nuclear gem counts it does not necessarily follow that an increase in nuclear gem counts must therefore reflect an increase in cellular SMN.

Recent literature reports have described a new role for DcpS that is suggestive of the mechanism of action of DAQ-DcpSi in SMA. In both yeast [30] and C.elegans [31], the orthologs of DcpS promote the exonucleolytic activity of Xrn1 and could therefore influence the stability of mRNA both directly and indirectly via altered miRNA levels. Some catalytically-inactive DcpS mutants can also promote Xrn1 activity, indicating that this effect is independent of enzyme activity of DcpS [31]. These observations have now been extended to human cells showing that DcpS regulates miRNA stability and that although this is a non-catalytic activity function of DcpS it is inhibited by DAQ-DcpSi, possibly due to allosteric modulation of the interaction with XRN2 [24].

We note that others [32] have reported that PAQR8 is modulated by RG3039 in a DcpS-independent manner based on the lack of effect of DcpS knockdown on the levels of this transcript. This is in marked contrast to our findings in which down-regulation of PAQR8 was both proportional to the degree of DcpS knockdown, and the ability of RG3039 to reduce PAQR8 transcripts was lost by 7-methyl substitution. It is possible that transcripts may exhibit differential sensitivities to DcpS knockdown, and that the degree of knockdown achieved by other investigators was insufficient to affect PAQR8 transcripts. Consistent with this, comparison of the Western blot data presented by these authors [32] and our own suggests that our clones 24 and 71 had a greater degree of knockdown than the DcpS^KD^ cells.

Overall, the effects of DAQ-DcpSi such as RG3039 on the cellular transcript profile are widespread and highly complex. To date, it is not clear why a given transcript is regulated by DAQ-DcpSi and of those transcripts that change there may be multiple mechanisms underlying their response, as RG3039 influences the stability of some transcripts that change but not others [32]. To some extent, the multitude of changes observed in DAQ-DcpSi-treated cells and tissues may reflect the choice of relatively long incubation/treatment times, and more detailed characterization of changes at early time points may help discriminate between primary effects and downstream sequelae.

DcpS was first identified as the putative target for DAQ compounds using a protein array of approximately 5,000 proteins [10]. We have also validated DcpS as the putative target of DAQ compounds in live cells using chemical probe and thermal stabilization assays [33]. Extending the work of Singh et al. to 9,000 proteins [34] has so far confirmed DcpS as the only identifiable binding partner of this class of compound to-date. Therefore, the diverse effects of DAQ-DcpSi may well reflect the breadth of effects of raising free m7G cap-containing species in cells, stabilizing miRNA and potentially other roles of DcpS that may not be fully understood currently.

## Conclusions

The effects of DAQ-DcpSi are subtle and complex and not consistent with direct activation of the *SMN2* promoter as expected from their mode of discovery. The compounds invoke a broad array of gene expression changes, with lack of bias in direction, and distribution across many pathways and functions. The data clearly support DcpS inhibition as the mode of action of the compounds, but the link between DcpS inhibition and improvement of disease phenotype in SMA mice is as yet unclear.

## Supporting information

S1 Fig Ingenuity pathway analysis on RG3039 differentially regulated genes. Ingenuity pathway analysis on RG3039 differentially regulated genes (corrected P value <0.05) ranked by—Log10(P) from Neuro2a cell RNA-seq analysis obtained from poly A RNA libraries.
(DOCX)

S2 Fig Ingenuity pathway analysis of diseases and bio function. (DOCX)

S3 Fig Pharmacokinetic characterization of PF-06738066 in FVB/N wild type mice. Brain, plasma and CSF PK of PF-06738066 in FVB/N mice following 30mg/kg IP administration.
(DOCX)

S4 Fig Pharmacokinetic profiling of PF-06738066 in P13 2B/- SMA and helathy littermate control pups. Brain and plasma exposure of PF-06738066 in P13 2B/- SMA and littermate control (2B/+) mice following 10mg/kg IP administration.
(DOCX)

S5 Fig Representative RNA and Western blot from in vivo 2B/- study. (DOCX)

S1 Table Lentiviral clones used for DcpS knockdown. (DOCX)

S2 Table Primer/ probe set used in ddPCR. (DOCX)

S3 Table Custom designed SMA taqman assays. (DOCX)

S4 Table Neuro2a cell RG3039 differentially regulated genes. (XLSX)

S5 Table 2B/- vehicle vs. 2B/+ vehicle RNASeq. (XLSX)

S6 Table Top 20 up_down regulated RNAs in vehicle 2B/- vs. vehicle 2B/+ spinal cord. (XLSX)

S7 Table 2B/- RG3039 vs. 2B/- vehicle RNASeq. (XLSX)

S8 Table Top 20 up_down RNAs in RG3039 2B/- vs. vehicle 2B/- spinal cord. (XLSX)

S9 Table 2B/+ RG3039 vs. vehicle RNASeq. (XLSX)

S10 Table 2B/- RG3039 vs 2B/+ vehicle RNASeq. (XLSX)

S11 Table RNAs in opposite direction in vehicle 2B/- vs. RG3039 treatment. (XLSX)
