# Supplementary material for: In vitro and in vivo effects of 2,4 diaminoquinazoline inhibitors of the decapping scavenger enzyme DcpS: Context-specific modulation of SMN transcript levels
Source: PLoS One. 2017 Sep 25;12(9):e0185079. doi: 10.1371/journal.pone.0185079 (PMC5612656; doi:10.1371/journal.pone.0185079)

**S1 Fig. Ingenuity Pathway Analysis on RG3039 differentially regulated genes.** Ingenuity pathway analysis on RG3039 differentially regulated genes (corrected P value <0.05) ranked by –Log10(P) from Neuro2a cell RNA-seq analysis obtained from poly A RNA libraries. (Enlarge image to view).


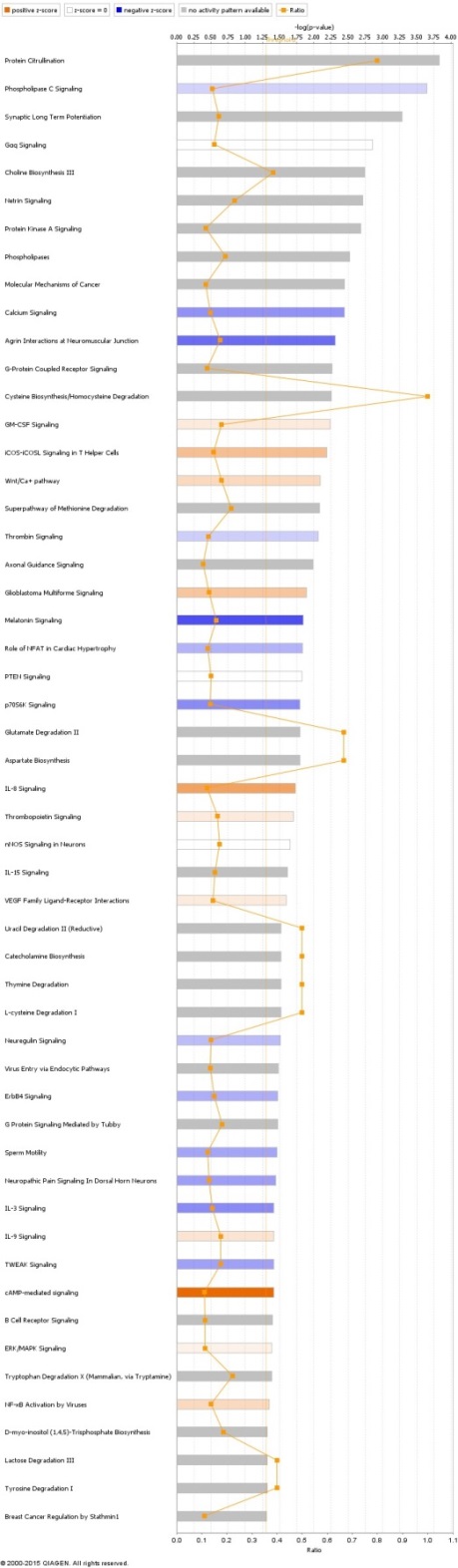

Supplement: S1 Fig — Ingenuity pathway analysis on RG3039 differentially regulated genes (corrected P value <0.05) ranked by—Log10(P) from Neuro2a cell RNA-seq analysis obtained from poly A RNA libraries. (DOCX) [file pone.0185079.s001.docx]
