# Supplementary material for: In vitro and in vivo effects of 2,4 diaminoquinazoline inhibitors of the decapping scavenger enzyme DcpS: Context-specific modulation of SMN transcript levels
Source: PLoS One. 2017 Sep 25;12(9):e0185079. doi: 10.1371/journal.pone.0185079 (PMC5612656; doi:10.1371/journal.pone.0185079)

**S2 Fig. Ingenuity Pathway analysis of diseases and Bio function.**

Ingenuity Pathway Analysis: Diseases and Bio Function analysis. (enlarge image to view)


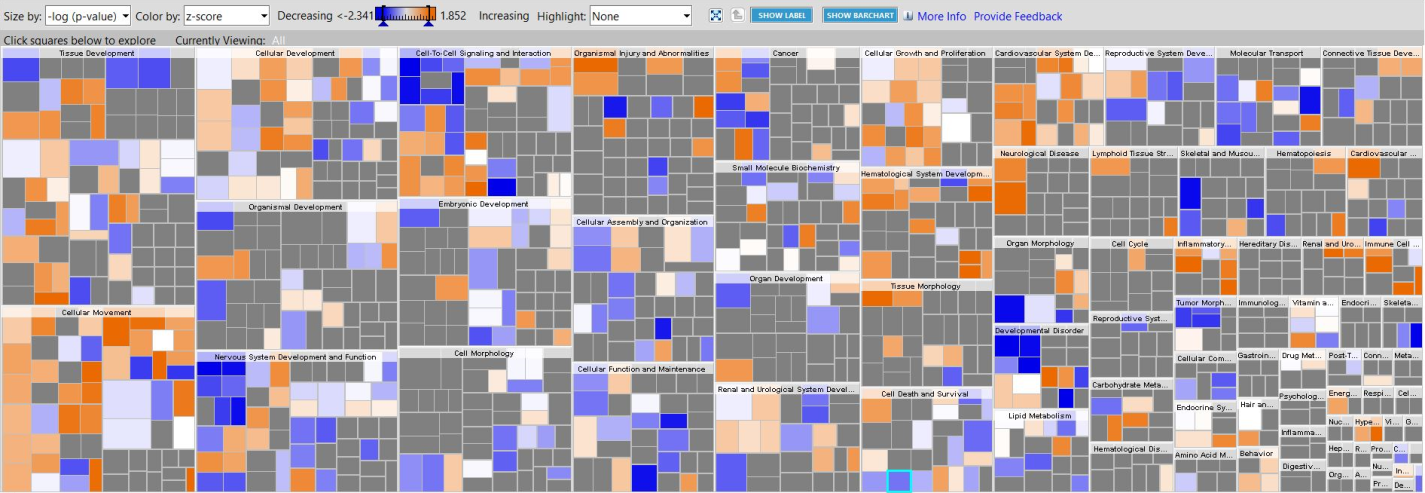

Supplement: S2 Fig — (DOCX) [file pone.0185079.s002.docx]
