# Supplementary material for: In vitro and in vivo effects of 2,4 diaminoquinazoline inhibitors of the decapping scavenger enzyme DcpS: Context-specific modulation of SMN transcript levels
Source: PLoS One. 2017 Sep 25;12(9):e0185079. doi: 10.1371/journal.pone.0185079 (PMC5612656; doi:10.1371/journal.pone.0185079)
Supplement: S3 Fig — Brain, plasma and CSF PK of PF-06738066 in FVB/N mice following 30mg/kg IP administration. (DOCX) [file pone.0185079.s003.docx]

**S3 Fig. Pharmacokinetic characterization of PF-06738066 in FVB/N wild type mice.** Brain, plasma and CSF PK of PF-06738066 in FVB/N mice following 30mg/kg IP administration.

| **PF-06738066** | **Cave ,u at 30mpk** |
| --- | --- |
| Plasma | 173 nM |
| Brain | 63 nM |
| CSF | 47 nM |
