# Supplementary material for: In vitro and in vivo effects of 2,4 diaminoquinazoline inhibitors of the decapping scavenger enzyme DcpS: Context-specific modulation of SMN transcript levels
Source: PLoS One. 2017 Sep 25;12(9):e0185079. doi: 10.1371/journal.pone.0185079 (PMC5612656; doi:10.1371/journal.pone.0185079)
Supplement: S4 Fig — Brain and plasma exposure of PF-06738066 in P13 2B/- SMA and littermate control (2B/+) mice following 10mg/kg IP administration. (DOCX) [file pone.0185079.s004.docx]

**S4 Fig.** **Pharmacokinetic profiling of PF-06738066 in P13 2B/- SMA and helathy littermate control pups.**  Brain and plasma exposure of PF-06738066 in P13 2B/- SMA and littermate control (2B/+) mice following 10mg/kg IP administration.


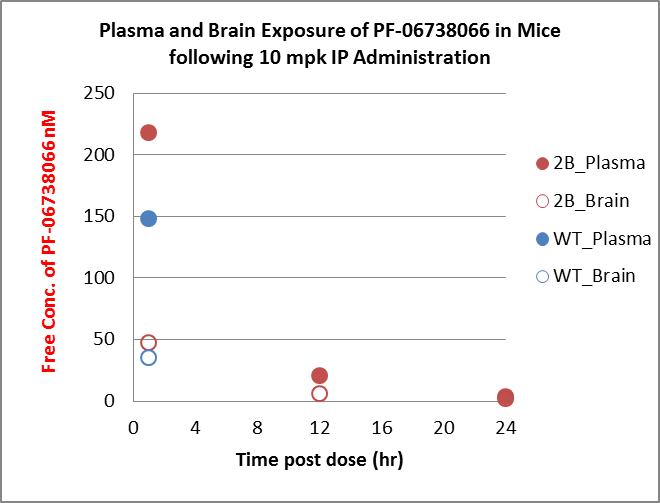
**A.**


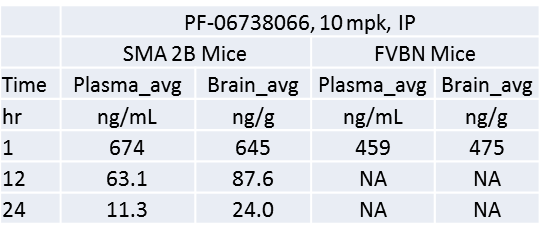
**B.**
