# Supplementary material for: In vitro and in vivo effects of 2,4 diaminoquinazoline inhibitors of the decapping scavenger enzyme DcpS: Context-specific modulation of SMN transcript levels
Source: PLoS One. 2017 Sep 25;12(9):e0185079. doi: 10.1371/journal.pone.0185079 (PMC5612656; doi:10.1371/journal.pone.0185079)

**S5 Fig. Representative RNA and western blot from in vivo 2B/- study.**

|  | **Expression level relative to PSMD14 in vehicle-treated 2B/- SMA mice** | | |
| --- | --- | --- | --- |
| **Gene** | **Spinal Cord** | **Skeletal muscle** | **Liver** |
| *Smn1* exon 7/8 | 0.0151 ± 0.0005 | 0.0069 ± 0.0005 | 0.0183 ± 0.0008 |
| *Dpm3* | 1.258 ± 0.015 | 0.704 ± 0.035 | 3.37 ± 0.07 |
| *Pigw* | 0.0180 ± 0.0005 | 0.0395 ± 0.0010 | 0.043 ± 0.002 |
| *Paqr8* | 0.119 ± 0.004 | 0.0025 ± 0.0012 | 0.0028 ± 0.0002 |

Representative Western blots from in vivo study


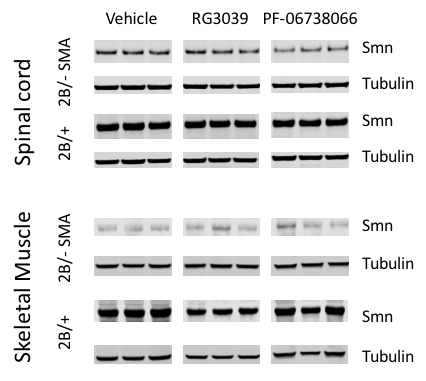

Supplement: S5 Fig — (DOCX) [file pone.0185079.s005.docx]
