# Supplementary material for: In vitro and in vivo effects of 2,4 diaminoquinazoline inhibitors of the decapping scavenger enzyme DcpS: Context-specific modulation of SMN transcript levels
Source: PLoS One. 2017 Sep 25;12(9):e0185079. doi: 10.1371/journal.pone.0185079 (PMC5612656; doi:10.1371/journal.pone.0185079)
Supplement: S1 Table — (DOCX) [file pone.0185079.s006.docx]

Table 1. Lentiviral clones used for DcpS knockdown (Sigma MISSION)

| **TRC# (Sigma MISSION)** | **Clone ID** | **Sequence** |
| --- | --- | --- |
| TRCN0000005570 | NM_014026.3-965s1c1 | CCGGGCTCGATGACTTGTACTTGATCTCGAGATCAAGTACAAGTCATCGAGCTTTTT |
| TRCN0000005571 | NM_014026.3-647s1c1 | CCGGGCAGTTCTCCAATGATATCTACTCGAGTAGATATCATTGGAGAACTGCTTTTT |
| TRCN0000335924 | NM_014026.3-1106s21c1 | CCGGGGGAGACCATCTGCGAGTATACTCGAGTATACTCGCAGATGGTCTCCCTTTTTG |
