# Supplementary material for: In vitro and in vivo effects of 2,4 diaminoquinazoline inhibitors of the decapping scavenger enzyme DcpS: Context-specific modulation of SMN transcript levels
Source: PLoS One. 2017 Sep 25;12(9):e0185079. doi: 10.1371/journal.pone.0185079 (PMC5612656; doi:10.1371/journal.pone.0185079)
Supplement: S2 Table — (DOCX) [file pone.0185079.s007.docx]

Table 2. Primer/probe sets used in ddPCR analysis

| **Single tube Taqman Assays (Life Technologies)** | |
| --- | --- |
| DcpS – FAM MGB | Hs00204009_m1 |
| DPM3 – FAM MGB | Hs00539663_s1 |
| PAQR8- FAM MGB | Hs01065322_s1 |
| PIGW- FMA MGB | Hs03028265_s1 |
| PPP4R2 – FAM MGB | Hs00752559_sH |
| PSMD14 – VIC MGB_PL | Hs01113429_m1 |
