# Supplementary material for: In vitro and in vivo effects of 2,4 diaminoquinazoline inhibitors of the decapping scavenger enzyme DcpS: Context-specific modulation of SMN transcript levels
Source: PLoS One. 2017 Sep 25;12(9):e0185079. doi: 10.1371/journal.pone.0185079 (PMC5612656; doi:10.1371/journal.pone.0185079)
Supplement: S3 Table — (DOCX) [file pone.0185079.s008.docx]

S3 Table. Custom designed SMA taqman assays (life Technoloiges)

| Gene | oligo | Sequence | Source |
| --- | --- | --- | --- |
| **SMN1** | Primer 1 (Fwd) | TACATGAGTGGCTATCATACTGGCTA | **Tiziano** et al., European Journal of Human Genetics, 2010, 18: 52-58 |
|  | Primer 2 (Rev) | AATGTGAGCACCTTCCTTCTTTTT |  |
|  | Probe (FAM-MGB-NFQ) | TATGGGTTT**C**AGACAAA |  |
| **SMN2** | Primer 1 (Fwd) | TACATGAGTGGCTATCATACTGGCTA |  |
|  | Primer 2 (Rev) | AATGTGAGCACCTTCCTTCTTTTT |  |
|  | Probe (FAM-MGB-NFQ) | ATATGGGTTT**T**AGACAAAA |  |
| **FL-SMNex7.8** | Primer 1 (Fwd) | AAAGAAGGAAGGTGCTCACATTC | **Simard** et al., *Neurology*, 2007, 68(6):451-6 |
|  | Primer 2 (Rev) | TGGTGTCATTTAGTGCTGCTCTATG |  |
|  | Probe (FAM-MGB-NFQ) | CAGCATTTCTCCTTAATTT |  |
| **Δ7-SMNex6.8** | Primer 1 (Fwd) | CATGGTACATGAGTGGCTATCATACTG |  |
|  | Primer 2 (Rev) | AGTGGTGTCATTTAGTGCTGCTCTAT |  |
|  | Probe (FAM-MGB-NFQ) | CCAGCATTTCCATATAATAG |  |
